# In-Depth Longitudinal Study of Listeria monocytogenes ST9 Isolates from the Meat Processing Industry: Resolving Diversity and Transmission Patterns Using Whole-Genome Sequencing

**DOI:** 10.1128/AEM.00579-20

**Published:** 2020-07-02

**Authors:** Annette Fagerlund, Solveig Langsrud, Trond Møretrø

**Affiliations:** a Nofima—Norwegian Institute of Food, Fisheries and Aquaculture Research, Ås, Norway; Centers for Disease Control and Prevention

**Keywords:** CC9, *Listeria monocytogenes*, ST9, WGS, food processing environment, food safety, meat industry, meat processing, whole-genome sequencing

## Abstract

Listeria monocytogenes is a pathogen mostly associated with the consumption of ready-to-eat foods and can cause severe disease and death. It can be introduced into food chains from raw materials, but often the contamination source is the food production environment, where certain clones can persist for years. In the meat chain, ST9 is one of the most commonly encountered L. monocytogenes sequence types, and for effective source tracking, the divergence and spread of ST9 must be understood. In this study, whole-genome sequencing (WGS) was used to characterize and track 252 L. monocytogenes ST9 isolates collected from four Norwegian meat processing plants between 2009 and 2017. The isolates formed distinct clusters relative to genomes found in public databases, and all but three isolates clustered into two major clonal populations. Different contamination patterns were revealed, e.g., evidence of contamination of two factories with a clone that diverged from its ancestor in the late 1990s through a common source of raw materials; breach of hygienic barriers within a factory, leading to repeated detection of two clones in the high-risk zone during a 4- to 6-year period; entry through the purchase and installation of second-hand equipment harboring a previously established clonal population; and spreading and diversification of two clones from two reservoirs within the same production room over a 9-year period. The present work provides data on the diversity of ST9, which is crucial for epidemiological investigations and highlights how WGS can be used for source tracking within food processing factories.

**IMPORTANCE** Listeria monocytogenes is a deadly foodborne pathogen that is widespread in the environment, and certain types can be established in food factories. The sequence type ST9 dominates in meat processing environments, and this work was undertaken to obtain data needed for the tracking of this subtype. By using whole-genome sequencing (WGS), we revealed the presence of cross-contamination routes between meat factories as well as within a single factory, including the spread from different reservoirs within the same room. It was also possible to estimate the time frame of persistence in the factory, as well as when and how new clones had entered. The present work contributes valuable information about the diversity of ST9 and exemplifies the potential power of WGS in food safety management, allowing the determination of relationships between strains both in an international context and locally between and within factories.

## Abstract (precis)

Listeria monocytogenes is a deadly foodborne pathogen that is widespread in the environment, and certain types can be established in food factories. The sequence type ST9 dominates in meat processing environments, and this work was undertaken to obtain data needed for the tracking of this subtype. By using whole-genome sequencing (WGS), we revealed the presence of cross-contamination routes between meat factories as well as within a single factory, including the spread from different reservoirs within the same room. It was also possible to estimate the time frame of persistence in the factory, as well as when and how new clones had entered. The present work contributes valuable information about the diversity of ST9 and exemplifies the potential power of WGS in food safety management, allowing the determination of relationships between strains both in an international context and locally between and within factories.

## INTRODUCTION

Listeria monocytogenes is a pathogenic bacterium causing the severe foodborne disease listeriosis, which is mostly linked to the consumption of ready-to-eat (RTE) food like fresh produce, soft cheeses, lightly processed fish products, and meat products, such as cooked sausages and cold cuts. In 2017 and 2018, the largest foodborne outbreak of listeriosis reported so far, with 1,060 confirmed cases and 216 deaths, was reported in South Africa and linked to the consumption of an RTE meat product (1). Several other listeriosis outbreaks also have been linked to RTE meat products (2, 3). The main route of contamination of L. monocytogenes to RTE food products is through cross-contamination from the processing environment (4, 5). Many studies have reported that clones of L. monocytogenes may establish and persist for years in processing environments, including in factories in the meat industry (5–15).

Isolates of L. monocytogenes can be subdivided into genetic groups, including different sequence types (STs) from multilocus sequence typing (MLST), in which several related STs comprise a central clonal complex (CC). CC9, which includes ST9, is a frequently detected clone in meat products and in meat industry environments; it is common in many countries and was ranked the fourth most common clone worldwide (16–22). For instance, CC9 was the second most prevalent clone isolated from foods in a study comprising 3,333 food isolates collected in France, and the majority of the CC9 isolates (66.4%) were from meat products (23). ST9 has also been shown to be the most frequently isolated (33%) L. monocytogenes type in Spanish meat processing plants, detected in 14 of 18 sampled plants (10). Several other studies also report ST9 contaminations in meat industry environments (12–15, 24, 25). For example, in a previous study (26), we subtyped a total of 680 L. monocytogenes isolates from Norwegian meat and salmon processing plants and found that 70% of the isolates from the meat industry (obtained mainly from two extensively sampled factories) belonged to ST9.

To better understand contamination routes within food industry environments and during foodborne outbreaks of L. monocytogenes, there is a need for more information about the genetic variation between environmental isolates over time, both within single facilities and between different processing plants. Whole-genome sequencing (WGS) analysis, employing either genomic MLST or single-nucleotide polymorphism (SNP) analyses, has become the gold standard for typing of bacteria. However, the majority of publicly available WGS data from L. monocytogenes environmental isolates are from inspections by authorities, often performed as part of outbreak investigations, resulting in a sampling bias for data deposited in public databases (27). Furthermore, a study examining the genetic distances between L. monocytogenes collected by the FDA during inspections in the United States during 1999 to 2017 covered WGS data for 5,321 isolates from 846 facilities (28); however, for 93% of the facilities included in the study, fewer than 20 isolates were collected (isolates per facility, median of 2 and range of 1 to 98). This suggests that the number of isolates collected by authorities from each facility is usually limited. In most studies where WGS-based strategies have been employed to investigate L. monocytogenes clones from food processing environments, only isolates from a single processing facility were considered, and only limited numbers of isolates belonging to the same cluster or strain were analyzed (12, 15, 24, 29–33). Thus, the description of contamination patterns is, in most cases, primarily based on looking at the occurrence of certain sequence types in different parts of the factory. For example, in a study that sampled L. monocytogenes from a meat establishment with slaughter, deboning, and meat processing units for 1 year, eight L. monocytogenes isolates were isolated and subjected to WGS. Five ST9 isolates, originating from both the slaughtering line and the meat processing units, formed a cluster whose maximum pairwise SNP distance was five, leading to the conclusion that the strains from the processing units originated from the slaughter line (24). Although this study used WGS to provide insight into contamination patterns, sampling was performed on a limited number of occasions (4 days during 1 year), resulting in a small number of obtained isolates, likely limiting insight into the genetic diversity of strains in the processing plant.

As an empirical practice used in outbreak investigations, it has been suggested that for L. monocytogenes, a threshold of fewer than 20 SNPs indicates an epidemiological link between isolates (28, 34). However, in contrast to that observed in typical point-source outbreaks, outbreaks caused by a population of persistent strains, which have had time to evolve in their environment, may have genetic diversity greater than 20 SNPs (35, 36). Additional evidence, such as epidemiological data and traceback evidence, as well as the phylogenetic tree topology, is, in any case, required to establish the contamination source during outbreaks (27, 37), as more than one facility may be home to the same clone of L. monocytogenes. This may occur, for example, in cases where suppliers of contaminated raw materials have more than one customer. A few WGS-based studies have considered the diversity of L. monocytogenes isolates across multiple food-associated facilities. For example, in a longitudinal study by Stasiewicz et al. (38), 175 isolates of L. monocytogenes sampled over 2 years in 30 retail delis were subjected to WGS. The results showed the presence of both persistent isolates within a deli and strains repeatedly introduced from external sources. The finding of almost identical isolates in different delis also indicated a common source for the introduction of strains. In a study by Morganti et al. (25), 108 L. monocytogenes isolates were obtained from 38 Parma ham production facilities, and isolates with pulsed-field gel electrophoresis (PFGE) profiles found in more than one facility (*n* = 69) were subjected to WGS analysis. Seven different STs were identified, of which the most prevalent was ST9 (*n* = 24), and three tight clusters, differing in only 1 or 2 core SNPs, were detected in more than one facility, suggesting interlinks between the establishments. However, in general, studies investigating the diversity of persistent clones found in more than one facility are lacking. To avoid misleading conclusions regarding contamination sources, increased knowledge about the diversity of closely related L. monocytogenes isolates in practical cases is needed.

In the present study, 252 isolates of L. monocytogenes ST9 from four Norwegian meat processing plants were subjected to WGS analysis to get further insight into the diversity, transmission patterns, and entry routes. The goals of the present study were to (i) apply whole-genome MLST (wgMLST), core genome MLST (cgMLST), and SNP analyses to assess the genetic diversity of the ST9 strains; (ii) determine whether certain genetic clusters of ST9 were factory specific, to what extent they were present in the same factory during extended periods of time, and how they related to isolates from other sources; and (iii) explore whether WGS analyses can be used to differentiate between contamination scenarios: hygienic breach between zones, dissemination of persistent listeria from a specific niche, or wide dissemination of a specific variant within a zone.

## RESULTS

### WGS of Norwegian ST9 isolates from meat processing industry.

A total of 252 L. monocytogenes ST9 isolates from four Norwegian meat processing plants were subjected to whole-genome sequencing (WGS) analysis. The four processing plants were not related; none belonged to the same company, and they were located in different geographic areas of Norway. The isolates were mainly from samples taken from floors, drains, or food processing equipment (26). The majority (*n* = 245) were collected from two plants, M1 and M4, over a period of 9 and 6 years, respectively (Table 1; see also Table S1 in the supplemental material). Isolates from 2017 were from the plants’ regular environmental hygiene monitoring programs. ST9 isolates had also been found in two less intensively sampled facilities (M5 and M7), and these were included for comparison. *In silico* MLST analysis confirmed that all isolates belonged to ST9, except one isolate (MF6279), which was technically not ST9, as it lacked the *bglA* gene.

**TABLE 1:** Overview of L. monocytogenes ST9 isolates subjected to WGS analysis

Plant	No. of isolates for each plant and yr^*a*^									Total
	2009	2010	2011	2012	2013	2014	2015	2016	2017	
M1	2	2	5	39	13	12			34	107
M4				35	18	74	10		1	138
M5					4					4
M7							3			3

a Isolates from plant M7 and isolates from 2017 are from the present study, and the remaining isolates were from Møretrø et al. (26).

### The Norwegian clones formed distinct clusters relative to public reference strains.

**FIG 1:**
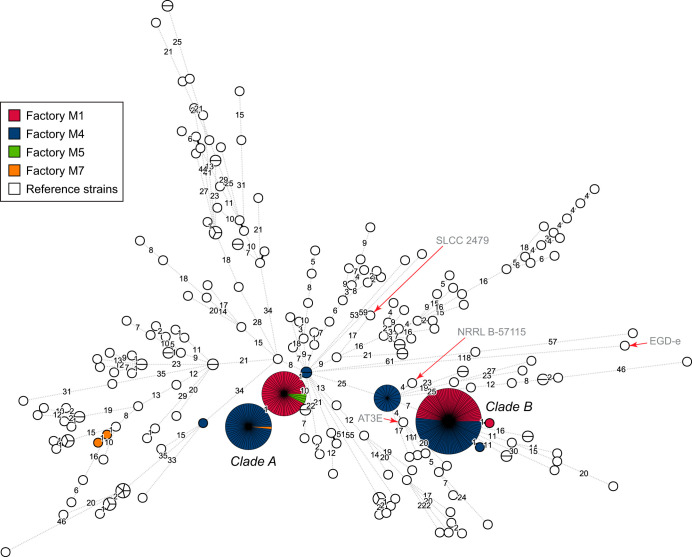
Minimum spanning tree based on cgMLST allelic profiles, showing the relationship between the 252 isolates from the Norwegian meat processing industry, colored according to the factory where they were isolated, and 202 CC9 reference genomes obtained from public databases (white nodes). The area of each circle is proportional to the number of isolates represented, and the number of allelic differences between isolates is indicated on the edges connecting two nodes. Labels clade A and clade B refer to the two main clusters of isolates from the Norwegian meat processing industry described in the text. Selected reference strains are indicated with red arrows.

To examine the relationship between the ST9 isolates from Norwegian meat processing plants and those from other sources, a representative subset of CC9 genomes found in public databases (*n* = 202) were selected for comparative analysis. A core genome MLST (cgMLST) analysis was performed using the subset of 1,748 cgMLST loci from the scheme described by Moura et al. (19). In this analysis, 88 polymorphic alleles were detected within the Norwegian data set, while a maximum of 160 allelic differences were identified between any pair from the 454 CC9 isolates included in the analysis. With the exception of three outliers, the Norwegian isolates clustered into two major genetic lineages, here referred to as clade A and clade B (Fig. 1). Isolates in these two clusters were separated by between 25 and 33 (median, 31) cgMLST alleles. Since the majority of the Norwegian isolates (*n* = 246) were represented by only four cgMLST profiles, three of which comprised isolates from two different meat processing plants, cgMLST typing was not sufficiently discriminative for the characterization of the diversity within the Norwegian data set. However, the analysis was sufficiently sensitive for differentiating between the Norwegian clones and those in the publicly available reference data set (Fig. 1). The reference strains most closely related to the Norwegian isolates were L. monocytogenes AT3E and NRRL B-57115, which were each separated by 3 or 4 alleles from isolates within clade B. AT3E was isolated from a food product in Finland in 1995 (39), while NRRL B-57115 (alias BCW_4316 [40]) was isolated from Italian roast beef in the United States. For the Norwegian clade A strains, the seven most closely related reference strains had 7 to 10 pairwise allelic differences compared to the closest Norwegian isolates. The two outlier strains MF6338 and MF6339, from plant M7, were separated by 1 and 2 cgMLST alleles from L. monocytogenes BCW_4274 (strain origin not indicated [40]). In conclusion, the Norwegian isolates in clades A and B were more related to each other than to any external/foreign strain from the cross-section of CC9 genomes from public databases.

### The ST9 isolates belong to two major clones.

**FIG 2:**
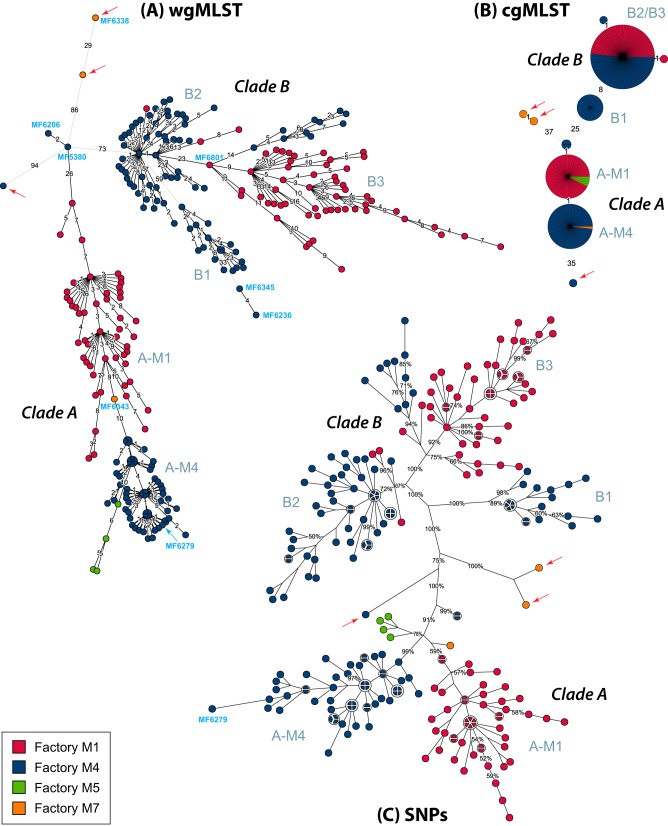
Phylogenetic trees for ST9 isolates from Norwegian meat processing industry. Shown are minimum spanning trees based on wgMLST (A) and cgMLST (B) analyses and a maximum parsimony tree based on an alignment of the 554 SNP positions detected using SLCC 2479 as the reference genome, showing the tree with the highest resampling support (C). Red arrows point to outliers. The area of each circle is proportional to the number of isolates represented, and the factory of origin for each isolate is indicated by the color. In panels A and B, branch labels indicate the numbers of target genes with differing alleles. In panel C, branch labels represent bootstrap resampling support values, showing only values of >50%. Branch lengths were scaled using logarithmic (A) and square root (B and C) scaling. Labels next to selected clusters refer to the subclusters described in the text. Selected isolate names are indicated in blue.

To differentiate between the Norwegian isolates, whole-genome MLST (wgMLST) and single-nucleotide polymorphism (SNP) analyses were performed. The wgMLST analysis detected a total of 3,334 loci, of which 746 were polymorphic, and between any pair of isolates a maximum of 179 (median, 95) allelic differences were found. As also observed from the cgMLST analysis, all but three of the isolates clustered into two major genetic lineages, clade A and clade B (Fig. 2A and B). Clade A includes isolates from all four investigated meat processing plants (*n* = 112), while only representatives from plants M1 and M4 were found in clade B (*n* = 137). Any pair of isolates within clade A differed by up to 59 (median, 26) wgMLST alleles, while the pairs of isolates in clade B showed a maximum of 129 (median, 34) allelic differences. The number of allelic differences separating isolates from clade A and clade B ranged from 73 to 165 (median, 119). With the exception of a subcluster of clade A isolates from plant M4, isolated during an intensively sampled 3-month period in 2014 (four groups comprising a total of 16 isolates), and two clade B isolates from 2015, all isolates could be distinguished using the wgMLST analysis.

The SNP analysis was performed by mapping raw reads from all 252 Norwegian isolates to the genome of the ST9 isolate L. monocytogenes SLCC 2479, for which a complete genome sequence is available (41). In addition, SNP analyses were performed separately for the isolates within clades A and B, using representatives from within each clade as reference genomes, MF4562 from clade A and MF4697 from clade B (42). Due to high sequence variability within prophage regions and plasmids, these regions were excluded from the SNP analyses. In the analysis performed for all genomes using SLCC 2479 as the reference (Fig. 2C), a total of 554 high-quality SNP positions were identified, of which 516 SNPs were present in all 252 isolates. The remaining 38 variant positions had missing data in up to 35% of the isolates, including a stretch of 23 consecutive SNP sites covering an ∼70-kb region, which included the missing *bglA* locus in MF6279. In comparison, of the 746 identified polymorphic wgMLST loci, only 494 were present in all isolates, reflecting the inclusion of accessory genes such as those found within prophages in the wgMLST scheme. In this SNP analysis, any pair of isolates differed by up to 123 (median, 58) variants while in the wgMLST analysis, they differed by up to 179 (median, 95) alleles, and a greater number of isolates remained undistinguishable in the SNP analysis than in wgMLST (Fig. 2). The number of SNP differences separating any pair of isolates from clade A and clade B ranged from 51 to 93 (median, 74), with 100% bootstrap support for the separation of clade A, clade B, and the three outlier strains.

The SNP analyses performed using representatives within clades A and B as reference genomes (Fig. S1) were more sensitive than the analysis performed using the external reference SLCC 2479 (Fig. 2C). A total of 121 SNPs were detected when reads from the clade A isolates were mapped to the MF4562 genome, compared to 106 SNPs detected using the SLCC 2479 reference. Any pair of isolates within clade A differed by up to 26 (median, 13) and 22 (median, 11) SNPs in the two analyses. Within clade B, the total numbers of detected SNPs were 331 and 287, and any pair of isolates differed by 77 (median, 27) and 64 (median, 24) SNPs, using MF4697 and SLCC 2479 as references, respectively. SNP counts reported below for strains within clade A or B are based on the analyses performed using MF4562 and MF4697 as reference genomes.

All three genomic analyses (wgMLST, cgMLST, and SNP) indicated that a subcluster within clade B comprising 19 isolates from plant M4, referred to as subcluster B1 (Fig. 2), could be considered a third major clade within this data set. This subgroup was separated from the remaining genomes in clade B with 100% bootstrap support (Fig. 2C and Fig. S1), between 45 and 77 (median, 61) SNPs, between 59 and 129 (median, 86) wgMLST alleles, and with 8 cgMLST alleles. Moura et al. (19) observed that isolates with no documented epidemiological link generally differed by more than ten cgMLST alleles. We conclude that a total of 249/252 (99%) of the L. monocytogenes ST9 isolates collected over a 9-year period (2009 to 2017) in four Norwegian meat processing plants belonged to one of two clonal lineages, referred to here as clades A and B.

### MLVA typing was not in accordance with the WGS phylogeny.

In previous work (26), the ST9 isolates collected between 2009 and 2015 were typed using the multiple-locus variable-number tandem repeat analysis (MLVA) scheme from Lindstedt et al. (43), also used by the Norwegian Institute of Public Health until March 2018. The majority of isolates belonged to three major MLVA profiles (26) (Table S1). The MLVA typing was, however, not fully concordant with the population structure determined using genomic analyses and was not sufficiently discriminatory to differentiate between isolates originating from different facilities (Fig. S2). This was not unexpected, since markers used in MLVA evolve by the addition or removal of repeat units. As a result, MLVA data comprise a substantial degree of phylogenetic noise (44).

### Spatiotemporal spread of ST9 clones within Norwegian meat supply chains.

**FIG 3:**
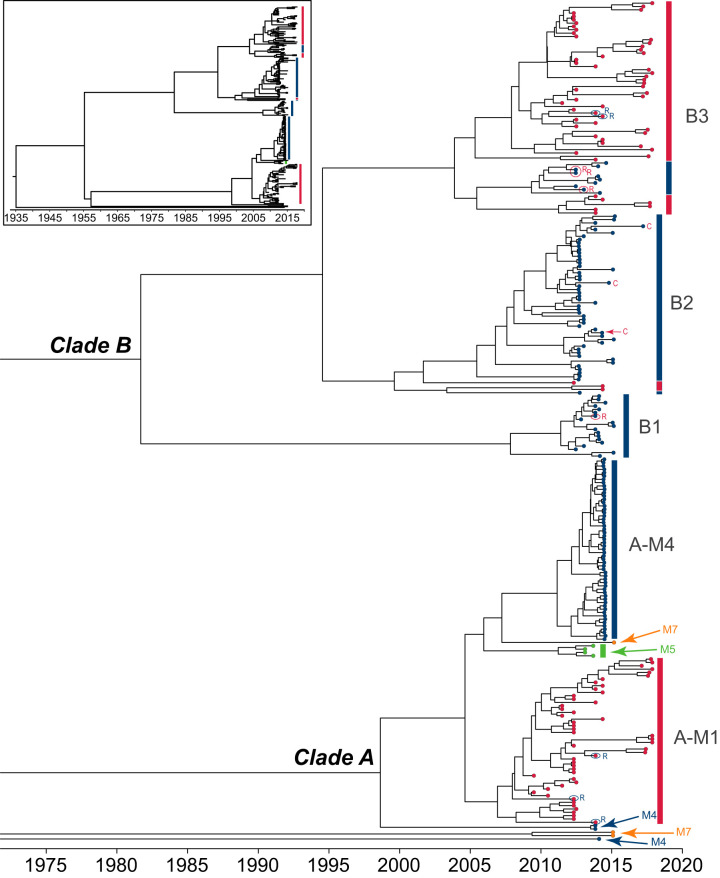
Time-scaled phylogenetic reconstruction, showing a maximum clade credibility tree produced from the concatenated SNP alignment obtained with SLCC 2479 as the reference genome, using the Bayesian Markov chain Monte Carlo method available in BEAST (45). The inset shows the whole tree, while the main figure shows the section containing the tips, enlarged for clarity. Figure S4 shows the same tree, labeled with the posterior probability values for each split and node labels containing strain names. Isolates within cluster B2 were isolated from raw zone sampling points, except for the three nodes labeled with C (cooked zone). All other isolates were from cooked zones, except the five nodes labeled with circles and an R (raw zone). The tips of the branches correspond to the sampling date, while tip colors indicate factory of origin for each strain, colored as described for Fig. 1 and Fig. 2. The scale is in years.

When the year of isolation was projected onto the wgMLST and SNP-based phylogenies (Fig. S3), no specific clustering of isolates according to isolation year was seen, except for subcluster A-M4, for which all isolates were obtained during 2014. To further investigate the dissemination and temporal history of the Norwegian ST9 isolates, a Bayesian tip-dated phylogenetic analysis (BEAST [45]) was conducted using the 554 differentiating SNPs, the date of isolation for each isolate, and assuming a strict molecular clock (Fig. 3 and Table 2 and Fig. S4). The analysis estimated that the most recent common ancestor (MRCA) of the set of 252 analyzed Norwegian ST9 isolates existed around the year 1935 (95% highest posterior density [HPD], 1907 to 1955; Table 2), while the division between the two main lineages (clades A and B) appeared to have occurred around 1955 (Fig. 3). The time of the MRCA was estimated to be 1998 for clade A (95% HPD, 1992 to 2004) and 1981 for clade B (95% HPD, 1970 to 1991). Since both clades contain isolates from multiple meat processing plants, cross-contamination between facilities, or contamination from a common primary source of L. monocytogenes, appears to have occurred in relatively recent years.

**TABLE 2:** Time of MRCA for the isolates in each clade estimated using BEAST analysis

Summary statistic	Mean time of MRCA^*a*^	95% HPD interval^*b*^	Mean SE	SD	ESS^*c*^
Age(root)	1934	1907.3, 1955.4	0.32	12.55	1,539
Age(cladeA)	1998	1991.8, 2004.0	0.06	3.22	3,212
Age(A-M1)	2007	2004.7, 2008.6	0.02	1.03	3,194
Age(A-M4)	2011	2009.2, 2012.5	0.01	0.89	3,956
Age(cladeB)	1981	1970.2, 1991.4	0.12	5.55	1,983
Age(B1)	2008	2004.1, 2011.1	0.03	1.88	4,796
Age(B2/B3)	1994	1987.0, 2000.6	0.07	3.56	2,294
Age(B2)	1999	1993.7, 2004.5	0.05	2.83	2,708
Age(B3)	2004	1999.9, 2007.2	0.04	1.93	2,173

a MRCA, most recent common ancestor.

b HPD, highest posterior density interval for the divergence time.

c ESS, effective sample size.

### Groups of isolates from all four factories were closely related.

The phylogenies based on the SNP analyses (Fig. 2C and 3 and Fig. S1 and S4) widely concur with the clustering of isolates obtained using wgMLST analysis (Fig. 2A), with incongruences in tree topology typically linked to branching points for outlier strains and smaller groups of isolates in clades with lower bootstrap and posterior probability support values. For instance, within clade A (comprising isolates from all four factories), the phylogenetic reconstructions based on the SNP matrices strongly support a monophyletic origin for a group of 55 isolates from plant M4 (named subcluster A-M4), presenting 99% bootstrap support (Fig. 2C and Fig. S1A), while the wgMLST analysis suggests that the factory M5 isolates belong to the same cluster as A-M4 (Fig. 2A). Support values for the branch delineating a second clade A subcluster, containing 50 isolates from plant M1 (named subcluster A-M1), as well as for the branches for the clade A isolates from factories M5 and M7, were low and/or showed conflicting results (Fig. 2C and Fig. S1A and S4). The pairwise distances between the factory M7 isolate (MF6343) and the isolates from factory M5, subclusters A-M1 and subcluster A-M4, ranged from 7 to 18 SNPs and 9 to 20 wgMLST loci. Therefore, although isolates within clade A largely clustered according to factory of origin, the small number of genetic differences between the groups indicated close genetic relationships between isolates from all four factories. Furthermore, the precise evolutionary relationship between these groups remains ambiguous. The BEAST analysis suggested that their time of divergence was approximately 10 to 15 years prior to the collection date for the most recently sampled isolates (Fig. 3).

### Clade B harbors clusters comprising isolates from both M1 and M4.

In clade B, both the wgMLST and SNP analyses show separation into three clearly delineated subclusters, named subclusters B1 (*n* = 19), B2 (*n* = 54), and B3 (*n* = 64) (Fig. 2 and Fig. S1B). Bootstrap support for the delineation of subclusters B1 and B3 from the remaining isolates in the tree was 100%, while support for the branch leading to subcluster B2 was lower (66% to 67%) (Fig. 2C and Fig. S1B), indicating that the grouping of cluster B2 was not as strongly supported. The cgMLST analysis was not able to differentiate between subclusters B2 and B3 (Fig. 2B). Subcluster B1 was estimated to have split from subclusters B2 and B3 around 1981, while the time of divergence of the B2 and B3 lineages was around 1994 (Table 2 lists 95% HPD intervals for the estimated times of MRCAs). Subcluster B1, with an MRCA estimated to have existed in 2008, exclusively contained isolates from only one factory (M4). Subclusters B2 and B3, however, both contained isolates from factories M1 and M4.

The subcluster B2 isolates from factories M1 (*n* = 3) and M4 (*n* = 51) were significantly associated with raw side production rooms (*P = *1.04 × 10^−37^ by two-sided Fisher’s exact test). Only three subcluster B2 isolates were from high-risk zones in which cooked meat was handled (in factory M4; see below), and subcluster B2 contained 51 of the 63 total isolates obtained from raw meat zones in the present study. The pairwise distances between the factory M1 and M4 isolates within subcluster B2 ranged from 8 to 24 SNPs and 12 to 23 wgMLST loci (median of 13 SNPs and 17 wgMLST loci), and the MRCA was estimated to have existed in 1999. The association of this subcluster with raw meat zones in both M1 and M4, along with the relatively close genetic relationship between isolates from both factories, indicates that for this clone the common link between the facilities is a contamination source related to raw materials.

Subcluster B3 contained 54 isolates from factory M1, most of them from the high-risk zone where cooked products were processed, plus a well-supported monophyletic cluster harboring ten factory M4 isolates (94% to 97% bootstrap support) (Fig. 2C and Fig. S1B). The pairwise distances between these factory M1 and M4 isolates ranged from 9 to 33 SNPs and 14 to 43 wgMLST loci (median of 21 SNPs and 26 wgMLST loci), and the MRCA was estimated to have existed in 2004. Thus, also within this subcluster, pairs of isolates from two different facilities had sufficiently close genetic relationships for a mistaken association of isolates to occur during a potential outbreak investigation, as a threshold genetic distance of <20 SNPs is commonly used to formulate a hypothesis that two isolates originate from the same source (28). The evidence of close association between isolates from more than one facility, within almost all branches of the phylogeny of L. monocytogenes ST9 from Norwegian meat processing facilities, further points to a widely interconnected meat industry sector in Norway.

### Related isolates in multiple areas of factory M4 suggests breach of hygienic barriers.

**FIG 4:**
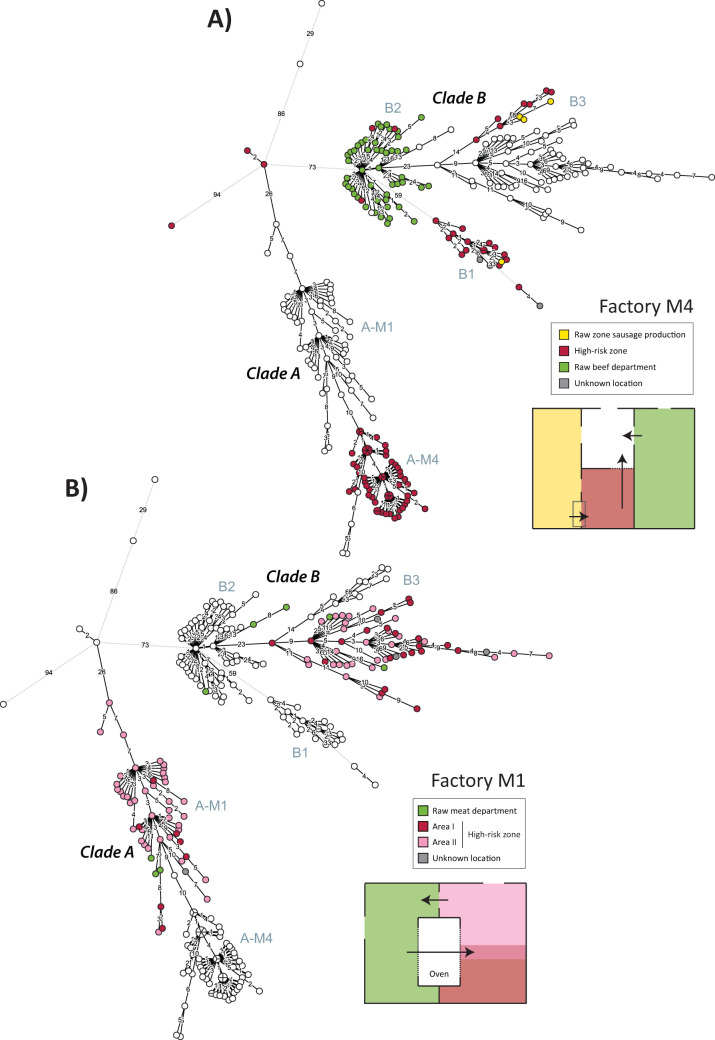
Minimum spanning trees based on wgMLST alleles, colored according to the location within factory M4 (A) and factory M1 (B) at which each L. monocytogenes isolate was obtained. Insets show sketches of the factory layouts, with arrows indicating the direction of movement of products and equipment through processing.

Factory M4 produces RTE meat products, like cold cuts, sausages, and liver pâté, as well as raw meat products, such as loin steaks and minced meat. The building structure houses two separate raw meat departments, one for sausage production and one for cutting and marinating raw beef. These departments are separated by hygienic barriers from the high-risk areas, where heat-treated products are processed and handled (Fig. 4A).

The high-risk zone and the raw sausage room harbored isolates belonging to subcluster B1 (*n* = 19, 2012 to 2015) and B3 (*n* = 10, 2012 to 2014) over a period of 3 to 4 years. None of these isolates appear to cluster in accordance with sampling area or the year of isolation (Fig. 3 and 4A and Fig. S3). The MRCA of the subcluster B1 isolates (for which isolates had a median pairwise distance of 3 SNPs [range, 0 to 14] and 5 wgMLST loci [range, 0 to 40]) was estimated to have existed around 2008 (Table 2), suggesting that this clone had persisted in the facility from around 2008 to 2015. The delineation of the M4 isolates within subcluster B3 (median pairwise distance, 8 SNPs [range, 0 to 14] and 9 wgMLST loci [range, 2 to 16]) had fairly good bootstrap support (94% to 97%; Fig. 2C and Fig. S1B), and the cluster had an estimated MRCA from about 2010 (Fig. 3), suggesting persistence for this clone from around 2010 to 2014. The analysis indicated that the breach of the hygienic barriers separating the raw sausage and high-risk zones had occurred on more than one occasion.

All the isolates from the second raw meat department in M4 (*n* = 48, 2012 to 2017) belonged to subcluster B2. Isolates from this subcluster were also found in three relatively recent samples (2014 and 2017) from the high-risk areas in the factory and formed a polyphyletic group within subcluster B2 (nodes labeled C in Fig. 3). The median pairwise distance between subcluster B2 isolates from M4 was 5 SNPs (range, 0 to 16) and 7 wgMLST loci (range, 1 to 18). These observations suggest that the three recent isolates originated from the raw beef cutting department, representing additional breaches of hygienic barriers in the factory.

In sum, the WGS data suggested that the clade B ST9 isolates found in the high-risk zone were related to those found in both adjacent departments handling raw meats. In 2016, as a response to these results, the factory decided to improve the hygienic barriers between departments to reduce contamination between the areas handling raw meat and the areas used for the processing of cooked products. Subsequently, only a single ST9 (subcluster B2) isolate was identified in the high-risk zone of factory M4 between January and October 2017 through the routine environmental monitoring program. The raw meat departments were not sampled after the reinforcement of hygienic barriers, but overall, the data suggest that the origin of the clade B ST9 isolates in factory M4 was in the raw meat departments.

### Contamination event related to installation of a second-hand slicer line.

The majority of the clade B isolates from factory M4 discussed above were from floors and drains. However, in 2014, factory M4 bought a used slicer line and installed it in the department where heat-treated products were processed. Within a week after starting production on the line, L. monocytogenes was detected in products (different types of sliced deli meats), product residues, and on processing equipment associated with the slicer line. The plant recalled products from the market after the first L. monocytogenes-positive samples. Despite several rounds with thorough cleaning and disinfection, including dismantling of the line, L. monocytogenes ST9 was repeatedly detected over the coming weeks. WGS analysis of the 55 ST9 isolates obtained in association with the introduction of the slicer line showed that they formed a separate and dense cluster within clade A, referred to as subcluster A-M4 (Fig. 2 and 4A). All isolates were closely related; a total of 41 SNP sites were identified, the median pairwise distance was 5 SNPs (range, 0 to 15) and 5 wgMLST loci (range, 0 to 12), and the MRCA of the A-M4 cluster was estimated to have existed in 2011 (Table 2). Isolates belonging to this subtype had not previously been detected in the factory.

After 8 weeks, the slicer line was discarded and completely removed from the processing plant, and the last subcluster A-M4 isolate was found 4 weeks after the slicer line was removed. Only three isolates obtained from the area of the factory where the line was installed, during the time it was present, were not part of subcluster A-M4 (they were typed as other STs). Thus, the WGS analysis confirmed that a persistent clone was introduced into the factory through the used slicer line. Similar cases of transfer of L. monocytogenes between factories have been documented in previous reports (8, 46). Most probably, the slicer line was contaminated with a population of closely related L. monocytogenes isolates when purchased, as the level of genetic variation between isolates was greater than what would be expected to develop within the short period the line was in use at plant M4. Unfortunately, the cluster could not be traced back to the factory where the slicer line originated, as this factory did not monitor L. monocytogenes in the processing environment, because they produced low-risk products (fermented meats) in which L. monocytogenes isolates are unlikely to grow.

### Two ST9 clones have diversified within the high-risk zone of factory M1.

Factory M1 produces various types of cooked sausages and pork liver pâté. The raw meat department is located next to a large high-risk production room used for processing and packaging of heat-treated products. The two departments are separated physically by large double-entry ovens as well as walls/doors and hygienic zone barriers (Fig. 4B). Sausages and other products enter the ovens from the raw side. After heat treatment, the oven doors on the cooked side are opened, and the products are taken into the high-risk production room. Racks used for carrying products inside the ovens are pushed back to the low-risk side after use. The majority of the isolates obtained from factory M1 (*n* = 107) were from two regions in the high-risk production room: an area for removing casings from sausages (*n* = 28; isolates were mainly from floors and drains) and an area containing conveyor-based packaging lines (*n* = 67; isolates were from floors, drains, and processing equipment/machines). The regions are referred to as area I and area II, respectively, in Fig. 4B. Although the areas had separate drains and equipment, flow of water, personnel, and trolleys could occur across the two regions, as they were not physically separated.

All isolates from the high-risk production room belonged to either subcluster A-M1 or subcluster B3. Subcluster A-M1 isolates (detected from 2009 to 2017) had a median distance between any pair of isolates of 4 SNPs (range, 0 to 12) and 11 wgMLST loci (range, 1 to 50), and the estimated time of existence of the MRCA was around 2007 (Table 2). For the subcluster B3 isolates from factory M1 (isolated from 2011 to 2017), the median pairwise distance was 11 SNPs (range, 0 to 27) and 16 wgMLST loci (range, 2 to 32), and the MRCA was estimated to have existed around 2004 (Table 2). Although isolates from both sampling areas I and II were found in both subclusters (as expected, since there were no barriers between these areas), isolates within subcluster B3 were more strongly associated with area I and the A-M1 isolates with area II (*P = *0.004 by two-sided Fisher’s exact test). There was, however, no clear pattern with respect to diversity between specific locations (Fig. 4B) and no apparent clustering of isolates according to isolation year (Fig. 3 and Fig. S3). The WGS results strongly indicated that the high-risk production room in factory M1 was home to two different ST9 lineages, both of which had diversified within the factory for a period of around ten years and were found in two distinct reservoirs (located in areas I and II) from which isolates were spread to other areas of the high-risk zone.

Only a few positive samples from plant M1 were obtained from the raw meat department (*n* = 8), as it was not part of the plant’s monitoring program for L. monocytogenes. Sampling was performed mainly in locations close to the high-risk zone at sites mentioned by factory personnel to have suboptimal hygienic zoning between the two departments. The ST9 isolates from the raw side were from three subclusters: A-M1 (*n* = 3), B3 (*n* = 2), and B2 (*n* = 3). Thus, five isolates belonged to the two ST9 subclusters found in the high-risk zone, indicating the occurrence of contamination between the two zones. Subcluster B2 isolates, in contrast, were not found in the more frequently sampled high-risk production room, suggesting that the direction of transmission of ST9 L. monocytogenes was more likely to be from the cooked to the raw side rather than *vice versa*.

## DISCUSSION

WGS was employed in the current study to investigate the diversity and transmission patterns of ST9 strains widely disseminated within the Norwegian meat processing industry and provides new insights into plant-to-plant and within-plant variation of L. monocytogenes. Several reports have shown that WGS may be used as a surveillance tool for tracking L. monocytogenes in food processing environments, and that high-resolution subtyping of bacterial isolates is necessary to unravel contamination routes within processing plants, as well as to track sources of foodborne outbreaks (24, 33, 34, 37, 47). The use of high-resolution WGS-based typing strategies is particularly appropriate for the investigation of widely distributed clones. However, many different bioinformatic methods can be used to analyze WGS data, and the various pipelines may not always yield concordant results (29, 48). In the current study, cgMLST did not yield sufficient resolution to differentiate between isolates originating from different processing plants, although it could separate the Norwegian clones from other publicly available CC9 reference genomes (Fig. 1). Furthermore, highly comparable results were obtained with the wgMLST- and SNP-based approaches (Fig. 2A and C; see also Fig. S1 in the supplemental material), largely grouping isolates according to their factory of origin. The wgMLST approach detected higher numbers of differentiating loci than the SNP analyses, even when SNP analysis was performed using reference genomes found within the same clusters as the examined isolates. Discrepancies between the two analyses were likely due to the exclusion of variable elements such as prophage and plasmid regions in the SNP analysis, while the employed wgMLST scheme includes accessory genes. The dependence on selection (or availability) of suitable reference genomes in SNP-based analyses may complicate interpretations of genetic distances between isolates. However, while the calculation of confidence support values for topologies of phylogenetic trees is straightforward for trees built using SNP analyses, it is less so for minimum spanning trees based on genomic MLST data (49). Confidence values should be taken into account when interpreting WGS analyses (27, 29).

The obtained results highlight the importance of considering evidence such as epidemiological data, traceback evidence, and phylogenetic tree topology, in addition to SNP counts, when seeking to establish potential sources of bacterial contaminations or outbreaks. The closest pairwise differences between any two isolates originating from two different facilities among the four examined in the current study ranged from 7 to 11 SNPs and from 9 to 11 wgMLST loci. These numbers are well below the threshold of 20 SNPs commonly employed during outbreak investigations as an indication that two isolates have originated in the same facility (28, 34). Our study also demonstrates the advantage of having a broad overview of the genotypes of strains circulating in food chains to prevent the misinterpretation of data during the investigation of contamination sources or in the event of potential outbreak scenarios. A more representative overview of circulating bacterial populations may both reveal the presence of closely related isolates present in multiple facilities and allow the detection of closely related but genetically distinct subpopulations through the building of more detailed phylogenies. A WGS-based analysis of a less deeply sampled data set than the ones currently considered (within clade A or B) could have resulted in the conclusion that isolates originated from the same source when in reality they belong to distinct subpopulations. The risk of misinterpretations would be even greater in the event that a potential outbreak was suspected of originating from a persistent clone, subject to diversification events within a factory, because in such cases the use of even more relaxed genetic distance thresholds has been recommended (35).

One of the questions posed in the current study was whether WGS could be used to differentiate between different contamination scenarios, including whether the presence of ST9 clones in an area of a factory was likely a result of persistence, multiple reintroduction events from an external source, or a combination of both. In the present work, the isolates formed five distinct subclusters, and for four of the clusters, closely related isolates were detected over the course of several (four to nine) years in the same meat processing facility. This typically indicates that the persistence of strains is the most common scenario. An alternative explanation is that isolates are more or less continuously introduced from an outside source in which the strain is persistent.

One clear indication that raw meat is a common source of the introduction of L. monocytogenes into more than one factory was the observation that subcluster B2 isolates, mainly found in the raw meat department used for cutting and marinating raw beef in factory M4, also were found on the raw side, but not in the high-risk zone, of factory M1. L. monocytogenes strains, including ST9 strains, have frequently been found to persist in meat slaughterhouses (5, 6, 15, 50–56). Therefore, a common primary source of a certain L. monocytogenes strain present in more than one meat processing plant potentially could be one that persists in a slaughterhouse supplying raw meat to multiple processing plants. The raw meat distribution chain in Norway is complex, where the same slaughterhouses deliver raw meat to many meat processing plants. The largest meat-producing company owns several slaughterhouses and sells raw meat to most meat processing plants. They also have a role as a market regulator and freeze meat for long-term storage during periods of high production. For beef, about 20% to 30% of the raw meat on the Norwegian market is imported, from lamb/mutton at 2% to 10% and <3% for pork and poultry, with some variation in import between years based on market demands (57). The M1 plant has its own slaughter department but also acquires raw meat from other slaughterhouses and sells raw meat to other processing plants, while M4 and M5 do not have slaughtering departments. Factory M7 has its own slaughter department for poultry but occasionally also buys raw poultry meat from others and routinely sells to other processing plants (>10 customers). Thus, it is evident that different factories may receive raw meat from the same supplier, which may explain why closely related isolates are found in multiple processing plants. The examination of L. monocytogenes isolates found upstream in the meat chain is needed to potentially identify the primary sources of the examined strains.

The primary origin of ST9 entering the food chain is not known. While L. monocytogenes is frequently found in animals, on farms, and in the natural environment, the frequency of the different genetic groups of L. monocytogenes appears to differ significantly in these environments compared to that observed in meat products and the meat processing industry. L. monocytogenes ST9 is rarely found in pig or ruminant farming environments, animals, or wild natural surroundings (22, 58–60). This sequence type has, however, frequently been found to dominate in raw pork meat (21, 52, 61) and is also commonly found to persist in slaughterhouses (14, 15, 24, 62) as well as in downstream compartments in the meat processing chain (10, 22, 26). In contrast, several studies report an absence or low abundance of ST9 in other food types and processing environments, such as fish/salmon, dairy, and fresh produce (22, 26, 31, 47, 63). These observations suggest that ST9 isolates are highly adapted to survival in meat production environments. We previously showed that an ST9 isolate included in the present study (MF4562, from subcluster A-M1) outcompeted isolates of other genotypes when grown in a meat-based growth medium (brain heart infusion [BHI]) in the presence of Listeria innocua (64). It also has been hypothesized that L. monocytogenes strains harboring *comK* prophages were adapted to meat environments (65), but information about the STs of examined strains was not provided. The genome data acquired in the current study will provide a basis for future studies aiming to determine potential underlying mechanisms responsible for the tight association of ST9 strains with the meat industry.

In conclusion, the current study confirms that isolates with few genetic differences were found across multiple meat processing plants and emphasizes the importance of having an overview of clones found in a given food chain to correctly interpret WGS analyses and prevent false-positive associations between isolates. The use of epidemiological data and traceback evidence is also fundamental for an accurate assessment of the origin of a given isolate. WGS analyses were shown to successfully distinguish between different contamination scenarios both between and within factories and can be used to estimate the time frame of persistence. Such information may be employed to significantly strengthen food safety management efforts within processing plants in the food industry.

## MATERIALS AND METHODS

### DNA isolation and whole-genome sequencing.

Bacteria were grown on BHI agar overnight at 37°C before a loopful of cells were suspended in 500 μl 2× Tris-EDTA (TE) buffer with 1.2% Triton X-100. Cells were lysed using lysing matrix B and a FastPrep instrument (both MP Biomedicals), and genomic DNA was isolated using the DNeasy blood and tissue kit (Qiagen). Libraries were prepared using the Nextera XT DNA sample preparation kit (Illumina) and sequenced either on a MiSeq platform with 300-bp paired-end (PE) reads or on a HiSeq platform with 150-bp PE reads (see Table S1 in the supplemental material). Sequencing on the HiSeq platform, and one of the MiSeq runs, were performed at the Norwegian Sequencing Centre (http://www.sequencing.uio.no); otherwise, sequencing was performed in-house. Raw reads were filtered on q15 and trimmed of adaptors using fastq-mcf from the ea-utils package (66).

### Genome assembly.

For 6 of the 252 isolates (MF4545, MF4562, MF4624, MF4697, MF4626, and MF6172), complete genome sequence assemblies were previously prepared using both Illumina and Nanopore sequencing data (42). For the remaining 246 isolates, *de novo* genome assemblies were generated using SPAdes v.3.10 (67) with the careful option and kmer sizes of 21, 33, 55, 77, 99, and 127. Contigs with sizes of <500 bp and kmer coverage of <5 were removed from the assemblies. The genome assemblies were evaluated using QUAST v2.2 (68).

### Genomic MLST analyses.

The wgMLST analysis was performed using a whole-genome schema containing 4,797 coding loci from the L. monocytogenes pangenome and the assembly-based BLAST approach, implemented in BioNumerics 7.6 (69). The cgMLST analysis was performed using the scheme described by Moura et al. (19), and results were either extracted from the wgMLST analysis (for isolates sequenced in the current study) or (for reference genomes) obtained using the KMA alignment method and the cgMLSTfinder program, which maps the filtered fastq reads to a database of the cgMLST alleles (70, 71). Minimum spanning trees were constructed using BioNumerics 7.6 based on the categorical differences in the allelic cgMLST or wgMLST profiles for each isolate. Loci with no allele calls were not considered in the pairwise comparison between two genomes. The number of allelic differences between isolates was read from genetic distance matrices computed from the absolute number of categorical differences between genomes.

### Selection of public reference genomes for comparison.

Reference genomes included in the cgMLST analysis of a cross-section of CC9 strains were selected and identified using the following sources. (i) Typing data were downloaded from the BIGSdb-*Lm* database (http://bigsdb.pasteur.fr/listeria/listeria.html) for the 19 L. monocytogenes sublineage 9 (SL9) isolates with associated cgMLST profiles publicly available on 19 March 2019. (ii) The L. monocytogenes AT3E genome (39) was selected for inclusion in the analysis, since it was previously identified as being related to isolates examined in the current study (42). As raw reads were not publicly available for AT3E, the cgMLST profile was obtained using the BioNumerics assembly-based wgMLST BLAST approach. For the remaining L. monocytogenes CC9 reference strains, publicly available raw fastq data were downloaded from the Sequence Read Archive (SRA) database (https://www.ncbi.nlm.nih.gov/sra). (iii) Of the 1,696 isolates included in the study by Moura et al. (19), 78 belonged to CC9, and of these, fastq read data were available for 55 genomes. cgMLST data for one strain (FSL R2-561) were also present in the BIGSdb data set; thus, 54 genomes were included. (iv) Among the 1,231 L. monocytogenes genome assemblies present in NCBI GenBank as of 6 December 2016, 87 were determined by *in silico* MLST analysis (72, 73) to belong to CC9, and of these, fastq read data were available for 27 isolates, of which nine were also present in the study by Moura et al. (19); thus, 18 genomes were obtained from this data set. (v) The LiSEQ project (74) included 110 L. monocytogenes CC9 strains from European countries that were included in the current analysis. In total, 202 CC9 reference genomes were selected for comparative analysis using cgMLST.

### SNP analysis.

The reference genomes used were L. monocytogenes SLCC 2479 (ST9; GenBank accession number NC_018589 [41]) and the chromosomes of L. monocytogenes MF4562 (clade A; GenBank accession number CP025442 [42]) and L. monocytogenes MF4697 (clade B, GenBank accession number CP025438 [42]). Each reference genome had two prophage regions, which were not conserved between strains and were excluded from the analysis. For each of the 252 ST9 isolates, Illumina reads filtered using fastq-mcf (described above) were mapped against the references using Bowtie2 v.2.2.6 or v.2.3.5.1 (75). Variants were called using FreeBayes v1.2.0-17 (76) with the ploidy option set to 1. The called variants were filtered using VCFtools (77) to remove indels, SNPs with quality values of <30, SNPs with missing data in >35% of genomes, and genotypes with <10 reads. SNPs clustered closer than 1,000 bp in the same individual isolate were excluded. The SNPs in each genome were extracted from the filtered FreeBayes outputs and concatenated into fasta pseudosequences containing only SNP sites (78), which then were used to generate multiple-sequence alignments. Maximum parsimony trees were built from the concatenated SNP alignments using BioNumerics 7.6 with default parameter values and 100× bootstrap resampling. In each analysis, the tree with the highest resampling support was computed. The SNP distance matrices summarizing the number of sites that differ between each pair of sequences were built from the filtered FreeBayes outputs using the dist.dna() function from the R package ape v.5.3 (79), using pairwise deletion of sites with missing data.

### Tip-dated phylogenetic analysis.

Timed phylogeny was reconstructed from the concatenated SNP alignment obtained with the SLCC 2479 reference genome, using the Bayesian Markov chain Monte Carlo (MCMC) method available in BEAST v1.10.4 (45). The sampling date for each isolate was entered as the tip date. For 21 isolates collected between 2009 and 2012, only the sampling year was known, and for these isolates, the sampling date was set to July 1 and the uncertainty value associated with the date was set to 1. For two isolates, only the sampling month and year were known, and for these, the 1st of the month was used. The most recently sampled isolate was sampled on 18 November 2017, which was set as the current date. To estimate the time of the most recent common ancestor (MRCA) for subclades, taxon sets were defined as comprising the isolates belonging to the clades and subclusters described in the study (clades A and B and subclusters A-M1, A-M4, B1, B2, B3, and B2/B3) (Table 2). Settings used were the HKY nucleotide substitution model, a strict molecular clock, and a coalescent constant size tree prior with a random starting tree. The analysis was run with a chain length of 10^9^, a sampling frequency of 10^5^, and a 5% burn-in. The effective sample size (ESS) values and trace files were monitored to ensure sufficient mixing (ESS of >1,000 for all statistics). The MCMC output was summarized as a maximum clade credibility tree, i.e., the tree with the highest product of the posterior probabilities of all its nodes.

### Data availability.

The raw reads have been deposited in the National Center for Biotechnology Information (NCBI) Sequence Read Archive (SRA) under BioProject accession no. PRJNA419519. Accession numbers for individual genomes have been submitted to NCBI's Sequence Read Archive and BioSample databases (accession numbers SRR11261968 to SRR11262219 and accession numbers SAMN08056483 to SAMN08056488 and SAMN14314680 to SAMN14314925; see Table S1 for details).

## Supplementary Material

Supplemental file 1
