# Supplementary material for: In-Depth Longitudinal Study of Listeria monocytogenes ST9 Isolates from the Meat Processing Industry: Resolving Diversity and Transmission Patterns Using Whole-Genome Sequencing
Source: Appl Environ Microbiol. 2020 Jul 2;86(14):e00579-20. doi: 10.1128/AEM.00579-20 (PMC7357480; doi:10.1128/AEM.00579-20)
Supplement: Supplemental file 1 [file AEM.00579-20-s0001.pdf]

SUPPLEMENTAL MATERIAL

In depth longitudinal study of *Listeria monocytogenes* ST9 isolates from meat processing industry: Resolving diversity and transmission patterns using WGS

Annette Fagerlund, Solveig Langsrud, Trond Møretrø

Nofima - Norwegian Institute of Food, Fisheries and Aquaculture Research, Ås, Norway

Supplemental Table S1: Details on sequencing, sample origin, and typing results for each isolate

Isolate	Instrument model	BioSample accession	SRA accession	No of filtered reads	Factory	Sampling date	Raw/cooked zone	Sampling area	MLVA profile	Clade	Subcluster
MF4536	Illumina MiSeq	SAMN14314680	SRR11262219	2 033 652	M1	27.04.2012	cooked	High-risk zone Area II	6-11-15-18-6	Clade A	A-M1
MF4537	Illumina MiSeq	SAMN14314681	SRR11262218	2 044 838	M1	27.04.2012	cooked	High-risk zone Area II	6-11-15-18-6	Clade A	A-M1
MF4538	Illumina MiSeq	SAMN14314682	SRR11262108	1 661 428	M1	27.04.2012	cooked	High-risk zone Area I	6-11-15-18-6	Clade A	A-M1
MF4539	Illumina MiSeq	SAMN14314683	SRR11262173	1 917 622	M1	27.04.2012	cooked	High-risk zone Area II	6-11-15-18-6	Clade A	A-M1
MF4540	Illumina MiSeq	SAMN14314684	SRR11262162	1 414 472	M1	27.04.2012	cooked	High-risk zone Area I	6-11-15-18-6	Clade A	A-M1
MF4541	Illumina MiSeq	SAMN14314685	SRR11262214	1 205 903	M1	27.04.2012	cooked	High-risk zone Area II	6-11-15-18-6	Clade A	A-M1
MF4542	Illumina MiSeq	SAMN14314686	SRR11262203	983 163	M1	27.04.2012	cooked	High-risk zone Area II	6-11-15-18-6	Clade A	A-M1
MF4543	Illumina MiSeq	SAMN14314687	SRR11262192	2 427 893	M1	27.04.2012	cooked	High-risk zone Area II	6-11-15-18-6	Clade A	A-M1
MF4544	Illumina MiSeq	SAMN14314688	SRR11262053	1 921 805	M1	27.04.2012	-	High-risk zone Area I	6-11-15-18-6	Clade A	A-M1
MF4545	Illumina MiSeq	SAMN08056483	SRR11262042	2 030 597	M1	27.04.2012	cooked	High-risk zone Area II	6-11-15-18-6	Clade A	A-M1
MF4546	Illumina MiSeq	SAMN14314689	SRR11262217	1 112 272	M1	27.04.2012	cooked	High-risk zone Area II	7-10-15-18-6	Clade B	B3
MF4547	Illumina MiSeq	SAMN14314690	SRR11262021	1 793 727	M1	27.04.2012	cooked	High-risk zone Area II	6-11-15-18-6	Clade A	A-M1
MF4549	Illumina MiSeq	SAMN14314691	SRR11262010	1 823 190	M1	27.04.2012	cooked	High-risk zone Area II	6-11-15-18-6	Clade A	A-M1
MF4550	Illumina MiSeq	SAMN14314692	SRR11262154	1 660 922	M1	27.04.2012	cooked	High-risk zone Area II	6-11-15-18-6	Clade A	A-M1
MF4551	Illumina MiSeq	SAMN14314693	SRR11262142	1 746 599	M1	27.04.2012	cooked	High-risk zone Area II	7-10-15-18-6	Clade B	B3
MF4552	Illumina MiSeq	SAMN14314694	SRR11262131	2 169 286	M1	27.04.2012	cooked	High-risk zone Area II	6-11-15-18-6	Clade A	A-M1
MF4553	Illumina MiSeq	SAMN14314695	SRR11261998	1 324 491	M1	27.04.2012	cooked	High-risk zone Area I	7-10-15-18-6	Clade B	B3
MF4554	Illumina MiSeq	SAMN14314696	SRR11261987	1 845 273	M1	27.04.2012	cooked	High-risk zone Area I	7-10-15-18-6	Clade B	B3
MF4555	Illumina MiSeq	SAMN14314697	SRR11261976	1 545 087	M1	27.04.2012	cooked	High-risk zone Area II	6-11-15-18-6	Clade A	A-M1
MF4556	Illumina MiSeq	SAMN14314698	SRR11262119	1 785 376	M1	27.04.2012	cooked	High-risk zone Area II	7-10-15-18-6	Clade B	B3
MF4557	Illumina MiSeq	SAMN14314699	SRR11262107	2 028 504	M1	27.04.2012	cooked	High-risk zone Area II	6-11-15-18-6	Clade A	A-M1
MF4558	Illumina MiSeq	SAMN14314700	SRR11262096	1 688 766	M1	27.04.2012	cooked	High-risk zone Area II	6-11-15-18-6	Clade A	A-M1
MF4559	Illumina MiSeq	SAMN14314701	SRR11262085	1 764 014	M1	27.04.2012	cooked	High-risk zone Area I	6-11-15-18-6	Clade A	A-M1
MF4560	Illumina MiSeq	SAMN14314702	SRR11262074	1 162 028	M1	27.04.2012	cooked	High-risk zone Area II	7-10-15-18-6	Clade B	B3
MF4561	Illumina HiSeq 3000	SAMN14314703	SRR11262063	2 838 409	M1	27.04.2012	cooked	High-risk zone Area II	6-11-15-18-6	Clade A	A-M1
MF4562	Illumina MiSeq	SAMN08056484	SRR11262180	2 044 412	M1	27.04.2012	raw	Raw meat department	6-11-15-18-6	Clade A	A-M1
MF4563	Illumina HiSeq 3000	SAMN14314704	SRR11262177	3 875 209	M1	27.04.2012	raw	Raw meat department	7-11-15-19-6	Clade B	B2
MF4624	Illumina MiSeq	SAMN08056485	SRR11262176	1 749 611	M4	18.06.2012	cooked	High-risk zone	7-11-15-18-6	Clade B	B3
MF4625	Illumina HiSeq 3000	SAMN14314705	SRR11262175	5 241 457	M4	18.06.2012	raw	Raw sausage room	7-11-15-18-6	Clade B	B3
MF4626	Illumina HiSeq 3000	SAMN08056486	SRR11262174	4 137 599	M4	18.06.2012	cooked	High-risk zone	7-11-15-18-6	Clade B	B1
MF4629	Illumina HiSeq 3000	SAMN14314706	SRR11262172	4 057 879	M4	18.06.2012	raw	Raw sausage room	7-11-15-18-6	Clade B	B3
MF4676	Illumina HiSeq 3000	SAMN14314707	SRR11262171	2 811 937	M4	30.08.2012	raw	Raw beef department	7-11-15-18-6	Clade B	B2
MF4677	Illumina HiSeq 3000	SAMN14314708	SRR11262170	3 822 090	M4	30.08.2012	raw	Raw beef department	7-11-15-18-6	Clade B	B2
MF4678	Illumina HiSeq 3000	SAMN14314709	SRR11262169	3 718 877	M4	30.08.2012	raw	Raw beef department	7-11-15-18-6	Clade B	B2
MF4679	Illumina HiSeq 3000	SAMN14314710	SRR11262168	2 403 646	M4	30.08.2012	raw	Raw beef department	7-11-15-18-6	Clade B	B2
MF4680	Illumina HiSeq 3000	SAMN14314711	SRR11262167	4 177 423	M4	03.09.2012	raw	Raw beef department	7-11-15-18-6	Clade B	B2
MF4681	Illumina HiSeq 3000	SAMN14314712	SRR11262166	2 448 580	M4	03.09.2012	raw	Raw beef department	7-11-15-18-6	Clade B	B2
MF4682	Illumina HiSeq 3000	SAMN14314713	SRR11262165	2 366 193	M4	03.09.2012	raw	Raw beef department	7-11-15-18-6	Clade B	B2
MF4685	Illumina HiSeq 3000	SAMN14314714	SRR11262164	2 573 444	M4	03.09.2012	raw	Raw beef department	7-11-15-18-6	Clade B	B2

MF4686	Illumina HiSeq 3000	SAMN14314715	SRR11262163	1 676 532	M4	03.09.2012	raw	Raw beef department	7-11-15-18-6	Clade B	B2
MF4687	Illumina HiSeq 3000	SAMN14314716	SRR11262161	2 258 693	M4	17.09.2012	raw	Raw beef department	7-11-15-18-6	Clade B	B2
MF4688	Illumina HiSeq 3000	SAMN14314717	SRR11262160	2 727 460	M4	17.09.2012	raw	Raw beef department	7-11-15-18-6	Clade B	B2
MF4689	Illumina HiSeq 3000	SAMN14314718	SRR11262159	2 287 260	M4	17.09.2012	raw	Raw beef department	7-11-15-18-6	Clade B	B2
MF4690	Illumina HiSeq 3000	SAMN14314719	SRR11262158	3 292 723	M4	17.09.2012	raw	Raw beef department	7-11-15-18-6	Clade B	B2
MF4691	Illumina HiSeq 3000	SAMN14314720	SRR11262157	3 535 205	M4	17.09.2012	raw	Raw beef department	7-11-15-18-6	Clade B	B2
MF4692	Illumina HiSeq 3000	SAMN14314721	SRR11262156	3 300 865	M4	17.09.2012	raw	Raw beef department	7-11-15-18-6	Clade B	B2
MF4693	Illumina HiSeq 3000	SAMN14314722	SRR11262155	3 199 547	M4	17.09.2012	raw	Raw beef department	7-11-15-18-6	Clade B	B2
MF4694	Illumina HiSeq 3000	SAMN14314723	SRR11262153	7 535 319	M4	17.09.2012	raw	Raw beef department	7-11-15-18-6	Clade B	B2
MF4695	Illumina HiSeq 3000	SAMN14314724	SRR11262216	2 163 052	M4	17.09.2012	raw	Raw beef department	7-11-15-18-6	Clade B	B2
MF4696	Illumina HiSeq 3000	SAMN14314725	SRR11262215	2 659 074	M4	17.09.2012	raw	Raw beef department	7-11-15-18-6	Clade B	B2
MF4697	Illumina MiSeq	SAMN08056487	SRR11262213	2 074 397	M4	17.09.2012	raw	Raw beef department	7-11-15-18-6	Clade B	B2
MF4994	Illumina MiSeq	SAMN14314726	SRR11262212	2 309 728	M4	14.01.2013	cooked	High-risk zone	6-11-15-18-6	Clade B	B1
MF4995	Illumina HiSeq 3000	SAMN14314727	SRR11262211	6 454 150	M1	01.07.2012	cooked	Unknown	6-10-15-18-6	Clade A	A-M1
MF4996	Illumina HiSeq 3000	SAMN14314728	SRR11262210	2 721 935	M1	01.07.2012	cooked	High-risk zone Area II	7-10-15-16-6	Clade B	B3
MF4997	Illumina HiSeq 3000	SAMN14314729	SRR11262209	2 706 796	M1	01.07.2012	cooked	High-risk zone Area I	7-10-15-18-6	Clade B	B3
MF4998	Illumina HiSeq 3000	SAMN14314730	SRR11262208	2 791 027	M1	01.07.2012	cooked	High-risk zone Area II	6-11-15-18-6	Clade A	A-M1
MF4999	Illumina HiSeq 3000	SAMN14314731	SRR11262207	2 494 020	M1	01.07.2009	cooked	High-risk zone Area II	6-11-15-18-6	Clade A	A-M1
MF5000	Illumina HiSeq 3000	SAMN14314732	SRR11262206	3 472 799	M1	01.07.2010	cooked	High-risk zone Area II	6-11-15-18-6	Clade A	A-M1
MF5001	Illumina HiSeq 3000	SAMN14314733	SRR11262205	3 201 999	M1	01.07.2011	cooked	High-risk zone Area II	6-11-15-18-6	Clade A	A-M1
MF5372	Illumina HiSeq 3000	SAMN14314734	SRR11262204	2 998 342	M5	11.09.2013	cooked	-	6-11-15-18-6	Clade A	-
MF5379	Illumina HiSeq 3000	SAMN14314735	SRR11262202	3 355 400	M4	05.11.2013	cooked	High-risk zone	7-11-15-18-6	Clade B	B3
MF5380	Illumina HiSeq 3000	SAMN14314736	SRR11262201	3 266 855	M4	05.11.2013	cooked	High-risk zone	6-11-15-18-6	Clade A	-
MF5383	Illumina HiSeq 3000	SAMN14314737	SRR11262200	2 441 874	M1	12.11.2013	cooked	High-risk zone Area II	7-11-15-18-6	Clade B	B3
MF5384	Illumina HiSeq 3000	SAMN14314738	SRR11262199	2 687 385	M1	12.11.2013	cooked	High-risk zone Area II	6-11-15-18-6	Clade A	A-M1
MF5386	Illumina HiSeq 3000	SAMN14314739	SRR11262198	3 017 584	M1	12.11.2013	cooked	High-risk zone Area II	7-10-15-18-6	Clade B	B3
MF5406	Illumina HiSeq 3000	SAMN14314740	SRR11262197	3 139 973	M4	14.01.2013	raw	Raw sausage room	7-11-15-18-6	Clade B	B3
MF5628	Illumina HiSeq 3000	SAMN14314741	SRR11262196	3 402 504	M5	12.02.2013	cooked	-	6-11-15-18-6	Clade A	-
MF5633	Illumina HiSeq 3000	SAMN14314742	SRR11262195	5 571 523	M1	13.05.2014	raw	Raw meat department	7-11-15-18-6	Clade B	B2
MF5635	Illumina HiSeq 3000	SAMN14314743	SRR11262194	4 050 689	M1	13.05.2014	cooked	High-risk zone Area I	7-10-15-18-6	Clade B	B3
MF5639	Illumina HiSeq 3000	SAMN14314744	SRR11262193	2 576 484	M4	11.02.2014	cooked	High-risk zone	7-11-16-18-6	outlier	-
MF5642	Illumina HiSeq 3000	SAMN14314745	SRR11262191	2 531 453	M4	29.04.2014	cooked	High-risk zone	7-11-15-18-6	Clade B	B1
MF5645	Illumina HiSeq 3000	SAMN14314746	SRR11262190	2 603 186	M1	13.05.2014	cooked	High-risk zone Area II	6-11-15-18-6	Clade A	A-M1
MF5648	Illumina HiSeq 3000	SAMN14314747	SRR11262189	2 419 134	M4	06.06.2014	cooked	High-risk zone	6-11-15-18-6	Clade A	A-M4
MF5649	Illumina HiSeq 3000	SAMN14314748	SRR11262188	3 094 056	M4	03.06.2014	cooked	High-risk zone	6-11-15-18-6	Clade A	A-M4
MF5653	Illumina HiSeq 3000	SAMN14314749	SRR11262187	3 379 255	M4	23.06.2014	cooked	High-risk zone	6-11-15-18-6	Clade A	A-M4
MF5655	Illumina HiSeq 3000	SAMN14314750	SRR11262186	3 808 507	M4	24.06.2014	cooked	High-risk zone	6-11-15-18-6	Clade A	A-M4
MF6152	Illumina HiSeq 3000	SAMN14314751	SRR11262057	5 450 371	M4	27.09.2012	raw	Raw beef department	7-11-15-18-6	Clade B	B2
MF6153	Illumina HiSeq 3000	SAMN14314752	SRR11262056	4 068 257	M4	27.09.2012	raw	Raw beef department	7-11-15-18-6	Clade B	B2
MF6154	Illumina HiSeq 3000	SAMN14314753	SRR11262055	3 920 314	M4	27.09.2012	raw	Raw beef department	7-11-15-18-6	Clade B	B2
MF6155	Illumina HiSeq 3000	SAMN14314754	SRR11262054	3 352 267	M4	27.09.2012	raw	Raw beef department	7-11-15-18-6	Clade B	B2

MF6156	Illumina HiSeq 3000	SAMN14314755	SRR11262052	4 557 836	M4	27.09.2012	raw	Raw beef department	7-11-15-18-6	Clade B	B2
MF6157	Illumina HiSeq 3000	SAMN14314756	SRR11262051	2 728 627	M4	27.09.2012	raw	Raw beef department	7-11-15-18-6	Clade B	B2
MF6158	Illumina HiSeq 3000	SAMN14314757	SRR11262050	2 936 433	M4	27.09.2012	raw	Raw beef department	7-11-15-18-6	Clade B	B2
MF6159	Illumina HiSeq 3000	SAMN14314758	SRR11262049	3 995 309	M4	27.09.2012	raw	Raw beef department	7-11-15-18-6	Clade B	B2
MF6160	Illumina HiSeq 3000	SAMN14314759	SRR11262048	2 985 521	M4	27.09.2012	raw	Raw beef department	7-11-15-18-6	Clade B	B2
MF6161	Illumina HiSeq 3000	SAMN14314760	SRR11262047	2 691 616	M4	27.09.2012	raw	Raw beef department	7-11-15-18-6	Clade B	B2
MF6162	Illumina HiSeq 3000	SAMN14314761	SRR11262046	1 329 084	M4	01.11.2012	-	Unknown	7-11-15-18-6	Clade B	B1
MF6166	Illumina HiSeq 3000	SAMN14314762	SRR11262045	2 755 094	M4	09.01.2013	raw	Raw beef department	7-11-15-18-6	Clade B	B2
MF6167	Illumina HiSeq 3000	SAMN14314763	SRR11262044	2 319 131	M4	09.01.2013	raw	Raw beef department	7-11-15-18-6	Clade B	B2
MF6168	Illumina HiSeq 3000	SAMN14314764	SRR11262043	2 932 714	M4	09.01.2013	raw	Raw beef department	7-11-15-18-6	Clade B	B2
MF6169	Illumina HiSeq 3000	SAMN14314765	SRR11262041	2 627 308	M4	09.01.2013	raw	Raw beef department	7-11-15-18-6	Clade B	B2
MF6170	Illumina HiSeq 3000	SAMN14314766	SRR11262040	3 853 177	M4	09.01.2013	raw	Raw beef department	7-11-15-18-6	Clade B	B2
MF6171	Illumina HiSeq 3000	SAMN14314767	SRR11262039	4 041 490	M5	12.02.2013	cooked	-	6-11-15-18-6	Clade A	-
MF6172	Illumina HiSeq 3000	SAMN08056488	SRR11262038	4 871 279	M1	01.07.2012	cooked	High-risk zone Area I	7-10-15-18-6	Clade B	B3
MF6173	Illumina HiSeq 3000	SAMN14314768	SRR11262037	3 929 306	M1	01.07.2012	cooked	High-risk zone Area II	7-10-15-18-6	Clade B	B3
MF6174	Illumina HiSeq 3000	SAMN14314769	SRR11262036	3 715 611	M1	01.07.2009	cooked	High-risk zone Area II	6-11-15-18-6	Clade A	A-M1
MF6175	Illumina HiSeq 3000	SAMN14314770	SRR11262035	3 268 255	M1	01.07.2010	cooked	High-risk zone Area II	6-11-15-18-6	Clade A	A-M1
MF6176	Illumina HiSeq 3000	SAMN14314771	SRR11262034	3 733 407	M1	01.07.2011	cooked	High-risk zone Area II	6-11-15-18-6	Clade A	A-M1
MF6177	Illumina HiSeq 3000	SAMN14314772	SRR11262033	4 468 248	M1	01.07.2011	cooked	High-risk zone Area I	6-11-15-18-6	Clade A	A-M1
MF6178	Illumina HiSeq 3000	SAMN14314773	SRR11262031	6 196 332	M1	01.07.2011	cooked	High-risk zone Area I	6-11-15-18-6	Clade B	B3
MF6179	Illumina HiSeq 3000	SAMN14314774	SRR11262032	3 111 873	M1	01.07.2011	cooked	High-risk zone Area II	6-11-15-18-6	Clade A	A-M1
MF6180	Illumina HiSeq 3000	SAMN14314775	SRR11262030	3 463 158	M1	01.07.2012	cooked	High-risk zone Area II	6-11-15-18-6	Clade B	B3
MF6181	Illumina HiSeq 3000	SAMN14314776	SRR11262029	2 478 233	M1	01.07.2012	cooked	High-risk zone Area II	6-11-15-18-6	Clade B	B3
MF6182	Illumina HiSeq 3000	SAMN14314777	SRR11262028	3 050 601	M1	01.07.2012	cooked	High-risk zone Area II	7-10-15-18-6	Clade B	B3
MF6183	Illumina HiSeq 3000	SAMN14314778	SRR11262027	2 896 389	M1	01.07.2012	cooked	High-risk zone Area II	7-10-15-18-6	Clade B	B3
MF6184	Illumina HiSeq 3000	SAMN14314779	SRR11262026	3 210 723	M1	01.07.2012	cooked	High-risk zone Area II	7-10-15-18-6	Clade B	B3
MF6185	Illumina HiSeq 3000	SAMN14314780	SRR11262025	2 767 030	M1	01.07.2012	cooked	High-risk zone Area II	7-10-15-18-6	Clade B	B3
MF6195	Illumina HiSeq 3000	SAMN14314781	SRR11262024	4 256 262	M5	11.09.2013	cooked	-	6-11-15-18-6	Clade A	-
MF6201	Illumina HiSeq 3000	SAMN14314782	SRR11262023	4 207 293	M4	05.11.2013	raw	Raw sausage room	7-11-15-18-6	Clade B	B1
MF6203	Illumina HiSeq 3000	SAMN14314783	SRR11262022	2 924 932	M4	05.11.2013	cooked	High-risk zone	7-11-15-18-6	Clade B	B1
MF6204	Illumina HiSeq 3000	SAMN14314784	SRR11262020	3 079 843	M4	05.11.2013	cooked	High-risk zone	7-11-15-18-6	Clade B	B1
MF6205	Illumina HiSeq 3000	SAMN14314785	SRR11262019	2 929 915	M4	05.11.2013	raw	Raw beef department	7-11-15-18-6	Clade B	B2
MF6206	Illumina HiSeq 3000	SAMN14314786	SRR11262018	4 577 733	M4	05.11.2013	cooked	High-risk zone	6-11-15-18-6	Clade A	-
MF6207	Illumina HiSeq 3000	SAMN14314787	SRR11262017	4 481 272	M4	05.11.2013	cooked	High-risk zone	7-11-15-18-6	Clade B	B1
MF6208	Illumina HiSeq 3000	SAMN14314788	SRR11262016	5 493 287	M4	05.11.2013	raw	Raw beef department	7-11-15-18-6	Clade B	B2
MF6209	Illumina HiSeq 3000	SAMN14314789	SRR11262015	7 202 684	M4	05.11.2013	raw	Raw beef department	7-11-15-18-6	Clade B	B2
MF6210	Illumina HiSeq 3000	SAMN14314790	SRR11262014	2 155 986	M4	05.11.2013	raw	Raw beef department	7-11-15-18-6	Clade B	B2
MF6211	Illumina HiSeq 3000	SAMN14314791	SRR11262013	2 924 167	M1	12.11.2013	raw	Raw meat department	6-11-15-18-6	Clade A	A-M1
MF6212	Illumina HiSeq 3000	SAMN14314792	SRR11262012	1 977 669	M1	12.11.2013	cooked	High-risk zone Area I	6-11-15-18-6	Clade A	A-M1
MF6213	Illumina HiSeq 3000	SAMN14314793	SRR11262011	2 199 141	M1	12.11.2013	cooked	High-risk zone Area II	7-11-15-18-6	Clade B	B3
MF6214	Illumina HiSeq 3000	SAMN14314794	SRR11262009	2 035 875	M1	12.11.2013	cooked	High-risk zone Area I	7-11-15-18-6	Clade B	B3

MF6215	Illumina HiSeq 3000	SAMN14314795	SRR11262008	1 965 525	M1	12.11.2013	cooked	High-risk zone Area I	7-10-15-18-6	Clade B	B3
MF6216	Illumina HiSeq 3000	SAMN14314796	SRR11262007	2 978 287	M1	12.11.2013	cooked	High-risk zone Area II	7-11-15-18-6	Clade B	B3
MF6217	Illumina HiSeq 3000	SAMN14314797	SRR11262006	3 084 659	M1	12.11.2013	cooked	High-risk zone Area II	6-11-15-18-6	Clade A	A-M1
MF6219	Illumina HiSeq 3000	SAMN14314798	SRR11262005	4 353 422	M1	12.11.2013	raw	Raw meat department	7-10-15-18-6	Clade B	B3
MF6220	Illumina HiSeq 3000	SAMN14314799	SRR11262004	4 251 541	M1	12.11.2013	raw	Raw meat department	6-11-15-18-6	Clade A	A-M1
MF6221	Illumina HiSeq 3000	SAMN14314800	SRR11262003	3 454 214	M1	12.11.2013	cooked	High-risk zone Area II	7-10-15-18-6	Clade B	B3
MF6222	Illumina HiSeq 3000	SAMN14314801	SRR11262002	3 162 588	M4	17.01.2014	cooked	High-risk zone	7-11-15-18-6	Clade B	B3
MF6223	Illumina HiSeq 3000	SAMN14314802	SRR11262001	3 435 500	M4	17.01.2014	cooked	High-risk zone	7-11-15-18-6	Clade B	B1
MF6224	Illumina HiSeq 3000	SAMN14314803	SRR11262000	3 641 283	M4	17.01.2014	cooked	High-risk zone	7-11-15-18-6	Clade B	B3
MF6225	Illumina HiSeq 3000	SAMN14314804	SRR11262152	4 385 144	M4	27.01.2014	cooked	High-risk zone	7-11-15-18-6	Clade B	B1
MF6226	Illumina HiSeq 3000	SAMN14314805	SRR11262151	4 032 149	M4	27.01.2014	cooked	High-risk zone	7-11-15-18-6	Clade B	B1
MF6227	Illumina HiSeq 3000	SAMN14314806	SRR11262150	2 818 918	M4	11.02.2014	cooked	Unknown	7-11-15-18-6	Clade B	B1
MF6228	Illumina HiSeq 3000	SAMN14314807	SRR11262149	1 834 462	M4	11.02.2014	cooked	High-risk zone	7-11-15-18-6	Clade B	B1
MF6229	Illumina HiSeq 3000	SAMN14314808	SRR11262148	1 835 611	M4	11.02.2014	cooked	High-risk zone	7-11-15-18-6	Clade B	B1
MF6234	Illumina HiSeq 3000	SAMN14314809	SRR11262147	1 920 296	M4	03.03.2014	cooked	High-risk zone	7-11-15-18-6	Clade B	B3
MF6235	Illumina HiSeq 3000	SAMN14314810	SRR11262146	2 328 664	M4	03.03.2014	cooked	High-risk zone	7-11-15-18-6	Clade B	B3
MF6236	Illumina HiSeq 3000	SAMN14314811	SRR11262145	2 547 382	M4	03.03.2014	cooked	Unknown	7-11-15-18-6	Clade B	B1
MF6237	Illumina HiSeq 3000	SAMN14314812	SRR11262144	2 800 915	M4	29.04.2014	cooked	High-risk zone	7-11-15-18-6	Clade B	B2
MF6238	Illumina HiSeq 3000	SAMN14314813	SRR11262143	3 317 068	M4	29.04.2014	raw	Raw beef department	7-11-15-18-6	Clade B	B2
MF6239	Illumina HiSeq 3000	SAMN14314814	SRR11262141	2 506 977	M4	29.04.2014	raw	Raw beef department	7-11-15-18-6	Clade B	B2
MF6240	Illumina HiSeq 3000	SAMN14314815	SRR11262140	2 778 852	M1	13.05.2014	cooked	High-risk zone Area II	7-10-15-18-6	Clade B	B3
MF6241	Illumina HiSeq 3000	SAMN14314816	SRR11262139	2 122 848	M1	13.05.2014	cooked	High-risk zone Area I	7-10-15-18-6	Clade B	B3
MF6242	Illumina HiSeq 3000	SAMN14314817	SRR11262138	3 335 344	M1	13.05.2014	cooked	High-risk zone Area II	7-10-15-18-6	Clade B	B3
MF6243	Illumina HiSeq 3000	SAMN14314818	SRR11262137	2 307 061	M1	13.05.2014	cooked	High-risk zone Area II	7-10-15-18-6	Clade A	A-M1
MF6244	Illumina HiSeq 3000	SAMN14314819	SRR11262136	3 683 255	M1	13.05.2014	raw	Raw meat department	7-10-15-18-6	Clade B	B2
MF6245	Illumina HiSeq 3000	SAMN14314820	SRR11262135	2 543 848	M1	13.05.2014	cooked	High-risk zone Area II	7-10-15-18-6	Clade A	A-M1
MF6246	Illumina HiSeq 3000	SAMN14314821	SRR11262134	4 298 042	M1	13.05.2014	cooked	High-risk zone Area I	7-10-15-18-6	Clade B	B3
MF6247	Illumina HiSeq 3000	SAMN14314822	SRR11262133	3 730 015	M1	13.05.2014	cooked	High-risk zone Area II	6-11-15-18-6	Clade A	A-M1
MF6248	Illumina HiSeq 3000	SAMN14314823	SRR11262132	3 568 228	M1	13.05.2014	raw	Raw meat department	7-10-15-18-6	Clade B	B3
MF6254	Illumina HiSeq 3000	SAMN14314824	SRR11262130	1 989 119	M4	06.06.2014	cooked	High-risk zone	6-11-15-18-6	Clade A	A-M4
MF6255	Illumina HiSeq 3000	SAMN14314825	SRR11262129	3 497 408	M4	06.06.2014	cooked	High-risk zone	6-11-15-18-6	Clade A	A-M4
MF6256	Illumina HiSeq 3000	SAMN14314826	SRR11262128	3 135 185	M4	06.06.2014	cooked	High-risk zone	6-11-15-18-6	Clade A	A-M4
MF6257	Illumina HiSeq 3000	SAMN14314827	SRR11262127	3 439 912	M4	06.06.2014	cooked	High-risk zone	6-11-15-18-6	Clade A	A-M4
MF6258	Illumina HiSeq 3000	SAMN14314828	SRR11262126	3 200 313	M4	12.06.2014	cooked	High-risk zone	6-11-15-18-6	Clade A	A-M4
MF6259	Illumina HiSeq 3000	SAMN14314829	SRR11262125	2 324 277	M4	12.06.2014	cooked	High-risk zone	6-11-15-18-6	Clade A	A-M4
MF6260	Illumina HiSeq 3000	SAMN14314830	SRR11262124	3 195 982	M4	12.06.2014	cooked	High-risk zone	6-11-15-18-6	Clade A	A-M4
MF6261	Illumina HiSeq 3000	SAMN14314831	SRR11262123	2 201 340	M4	03.06.2014	cooked	High-risk zone	6-11-15-18-6	Clade A	A-M4
MF6262	Illumina HiSeq 3000	SAMN14314832	SRR11262122	2 205 014	M4	03.06.2014	cooked	High-risk zone	6-11-15-18-6	Clade A	A-M4
MF6263	Illumina HiSeq 3000	SAMN14314833	SRR11261999	2 454 772	M4	01.06.2014	cooked	High-risk zone	6-11-15-18-6	Clade A	A-M4
MF6265	Illumina HiSeq 3000	SAMN14314834	SRR11261997	2 334 798	M4	23.06.2014	cooked	High-risk zone	6-11-15-18-6	Clade A	A-M4
MF6267	Illumina HiSeq 3000	SAMN14314835	SRR11261996	2 778 658	M4	23.06.2014	cooked	High-risk zone	6-11-15-18-6	Clade A	A-M4

MF6268	Illumina HiSeq 3000	SAMN14314836	SRR11261995	2 668 700	M4	23.06.2014	cooked	High-risk zone	6-11-15-18-6	Clade A	A-M4
MF6270	Illumina HiSeq 3000	SAMN14314837	SRR11261994	1 468 027	M4	23.06.2014	cooked	High-risk zone	6-11-15-18-6	Clade A	A-M4
MF6272	Illumina HiSeq 3000	SAMN14314838	SRR11261993	2 115 690	M4	23.06.2014	cooked	High-risk zone	6-11-15-18-6	Clade A	A-M4
MF6273	Illumina HiSeq 3000	SAMN14314839	SRR11261992	2 288 118	M4	23.06.2014	cooked	High-risk zone	6-11-15-18-6	Clade A	A-M4
MF6275	Illumina HiSeq 3000	SAMN14314840	SRR11261991	3 158 856	M4	23.06.2014	cooked	High-risk zone	6-11-15-18-6	Clade A	A-M4
MF6276	Illumina HiSeq 3000	SAMN14314841	SRR11261990	3 015 680	M4	23.06.2014	cooked	High-risk zone	6-11-15-18-6	Clade A	A-M4
MF6278	Illumina HiSeq 3000	SAMN14314842	SRR11261989	3 276 231	M4	23.06.2014	cooked	High-risk zone	6-11-15-18-6	Clade A	A-M4
MF6279	Illumina HiSeq 3000	SAMN14314843	SRR11261988	4 509 460	M4	23.06.2014	cooked	High-risk zone	6-11-15-18-6	Clade A	A-M4
MF6281	Illumina HiSeq 3000	SAMN14314844	SRR11261986	3 019 582	M4	23.06.2014	cooked	High-risk zone	6-11-15-18-6	Clade A	A-M4
MF6283	Illumina HiSeq 3000	SAMN14314845	SRR11261985	2 849 259	M4	24.06.2014	cooked	High-risk zone	6-11-15-18-6	Clade A	A-M4
MF6284	Illumina HiSeq 3000	SAMN14314846	SRR11261984	7 017 732	M4	24.06.2014	cooked	High-risk zone	6-11-15-18-6	Clade A	A-M4
MF6285	Illumina HiSeq 3000	SAMN14314847	SRR11261983	3 234 276	M4	24.06.2014	cooked	High-risk zone	6-11-0-18-0	Clade A	A-M4
MF6286	Illumina HiSeq 3000	SAMN14314848	SRR11261982	2 758 884	M4	24.06.2014	cooked	High-risk zone	6-11-15-18-6	Clade A	A-M4
MF6287	Illumina HiSeq 3000	SAMN14314849	SRR11261981	2 027 285	M4	24.06.2014	cooked	High-risk zone	6-11-15-18-6	Clade A	A-M4
MF6289	Illumina HiSeq 3000	SAMN14314850	SRR11261980	3 175 952	M4	24.06.2014	cooked	High-risk zone	6-11-15-18-6	Clade A	A-M4
MF6290	Illumina HiSeq 3000	SAMN14314851	SRR11261979	3 258 902	M4	24.06.2014	cooked	High-risk zone	6-11-15-18-6	Clade A	A-M4
MF6291	Illumina HiSeq 3000	SAMN14314852	SRR11261978	5 637 788	M4	30.06.2014	cooked	High-risk zone	6-11-15-18-6	Clade A	A-M4
MF6292	Illumina HiSeq 3000	SAMN14314853	SRR11261977	2 950 993	M4	30.06.2014	cooked	High-risk zone	6-11-15-18-6	Clade A	A-M4
MF6294	Illumina HiSeq 3000	SAMN14314854	SRR11261975	2 960 406	M4	30.06.2014	cooked	High-risk zone	6-11-15-18-6	Clade A	A-M4
MF6295	Illumina HiSeq 3000	SAMN14314855	SRR11261974	2 732 601	M4	30.06.2014	cooked	High-risk zone	6-11-15-18-6	Clade A	A-M4
MF6296	Illumina HiSeq 3000	SAMN14314856	SRR11261973	3 991 404	M4	30.06.2014	cooked	High-risk zone	6-11-15-18-6	Clade A	A-M4
MF6297	Illumina HiSeq 3000	SAMN14314857	SRR11261972	2 963 795	M4	30.06.2014	cooked	High-risk zone	6-11-15-18-6	Clade A	A-M4
MF6298	Illumina HiSeq 3000	SAMN14314858	SRR11261971	3 274 724	M4	30.06.2014	cooked	High-risk zone	6-11-15-18-6	Clade A	A-M4
MF6299	Illumina HiSeq 3000	SAMN14314859	SRR11261970	2 357 811	M4	30.06.2014	cooked	High-risk zone	6-11-15-18-6	Clade A	A-M4
MF6300	Illumina HiSeq 3000	SAMN14314860	SRR11261969	2 939 662	M4	18.07.2014	cooked	High-risk zone	6-11-15-18-6	Clade A	A-M4
MF6301	Illumina HiSeq 3000	SAMN14314861	SRR11261968	2 467 577	M4	17.07.2014	cooked	High-risk zone	6-11-15-18-6	Clade A	A-M4
MF6302	Illumina HiSeq 3000	SAMN14314862	SRR11262121	2 548 215	M4	17.07.2014	cooked	High-risk zone	6-11-15-18-6	Clade A	A-M4
MF6303	Illumina HiSeq 3000	SAMN14314863	SRR11262120	2 666 079	M4	18.07.2014	cooked	High-risk zone	6-11-15-18-6	Clade A	A-M4
MF6304	Illumina HiSeq 3000	SAMN14314864	SRR11262118	3 276 383	M4	23.07.2014	cooked	High-risk zone	6-11-15-18-6	Clade A	A-M4
MF6306	Illumina HiSeq 3000	SAMN14314865	SRR11262117	3 510 331	M4	23.07.2014	cooked	High-risk zone	6-11-15-18-6	Clade A	A-M4
MF6307	Illumina HiSeq 3000	SAMN14314866	SRR11262116	3 865 116	M4	23.07.2014	cooked	High-risk zone	6-11-15-18-6	Clade A	A-M4
MF6308	Illumina HiSeq 3000	SAMN14314867	SRR11262115	4 594 495	M4	23.07.2014	cooked	High-risk zone	7-11-15-18-6	Clade B	B1
MF6309	Illumina HiSeq 3000	SAMN14314868	SRR11262114	5 258 714	M4	24.07.2014	cooked	High-risk zone	6-11-15-18-6	Clade A	A-M4
MF6310	Illumina HiSeq 3000	SAMN14314869	SRR11262113	2 375 622	M4	24.07.2014	cooked	High-risk zone	6-11-15-18-6	Clade A	A-M4
MF6311	Illumina HiSeq 3000	SAMN14314870	SRR11262112	4 341 626	M4	25.07.2014	cooked	High-risk zone	6-11-15-18-6	Clade A	A-M4
MF6312	Illumina HiSeq 3000	SAMN14314871	SRR11262111	3 193 262	M4	25.07.2014	cooked	High-risk zone	6-11-15-18-6	Clade A	A-M4
MF6313	Illumina HiSeq 3000	SAMN14314872	SRR11262110	2 307 762	M4	07.07.2014	cooked	High-risk zone	6-11-15-18-6	Clade A	A-M4
MF6316	Illumina HiSeq 3000	SAMN14314873	SRR11262109	3 846 459	M4	08.07.2014	cooked	High-risk zone	6-11-15-18-6	Clade A	A-M4
MF6317	Illumina HiSeq 3000	SAMN14314874	SRR11262106	3 103 656	M4	08.07.2014	cooked	High-risk zone	6-11-15-18-6	Clade A	A-M4
MF6318	Illumina HiSeq 3000	SAMN14314875	SRR11262105	4 402 013	M4	08.07.2014	cooked	High-risk zone	6-11-15-18-6	Clade A	A-M4
MF6320	Illumina HiSeq 3000	SAMN14314876	SRR11262104	2 216 619	M4	11.08.2014	cooked	High-risk zone	7-11-15-18-6	Clade B	B3

MF6324	Illumina HiSeq 3000	SAMN14314877	SRR11262103	2 068 422	M4	22.10.2014	cooked	High-risk zone	7-11-15-18-6	Clade B	B2
MF6329	Illumina HiSeq 3000	SAMN14314878	SRR11262102	2 412 500	M4	03.02.2015	raw	Raw beef department	7-11-15-18-6	Clade B	B2
MF6330	Illumina HiSeq 3000	SAMN14314879	SRR11262101	3 184 663	M4	03.02.2015	raw	Raw beef department	7-11-15-18-6	Clade B	B2
MF6332	Illumina HiSeq 3000	SAMN14314880	SRR11262100	3 039 897	M4	03.02.2015	raw	Raw beef department	7-11-15-18-6	Clade B	B2
MF6334	Illumina HiSeq 3000	SAMN14314881	SRR11262099	3 670 560	M4	28.01.2015	raw	Raw beef department	7-11-15-18-6	Clade B	B2
MF6337	Illumina HiSeq 3000	SAMN14314882	SRR11262098	3 253 085	M4	28.01.2015	cooked	High-risk zone	7-11-15-18-6	Clade B	B1
MF6338	Illumina HiSeq 3000	SAMN14314883	SRR11262097	4 411 005	M7	03.02.2015	raw	-	8-10-15-18-6	outlier	-
MF6339	Illumina HiSeq 3000	SAMN14314884	SRR11262095	2 372 262	M7	03.02.2015	raw	-	7-10-15-18-6	outlier	-
MF6343	Illumina HiSeq 3000	SAMN14314885	SRR11262094	2 680 958	M7	03.03.2015	raw	-	6-11-15-18-6	Clade A	-
MF6345	Illumina HiSeq 3000	SAMN14314886	SRR11262093	2 840 681	M4	17.02.2015	cooked	High-risk zone	7-11-15-18-6	Clade B	B1
MF6346	Illumina HiSeq 3000	SAMN14314887	SRR11262092	2 531 134	M4	24.02.2015	raw	Raw beef department	7-11-15-18-6	Clade B	B2
MF6348	Illumina HiSeq 3000	SAMN14314888	SRR11262091	3 455 548	M4	24.02.2015	cooked	High-risk zone	7-11-15-18-6	Clade B	B1
MF6350	Illumina HiSeq 3000	SAMN14314889	SRR11262090	5 608 298	M4	03.03.2015	raw	Raw beef department	7-11-15-18-6	Clade B	B2
MF6356	Illumina HiSeq 3000	SAMN14314890	SRR11262089	4 047 199	M4	24.03.2015	raw	Raw beef department	7-11-15-18-6	Clade B	B2
MF6577	Illumina MiSeq	SAMN14314891	SRR11262088	1 239 738	M1	23.01.2017	cooked	High-risk zone Area I	-	Clade B	B3
MF6578	Illumina MiSeq	SAMN14314892	SRR11262087	995 301	M1	25.01.2017	cooked	High-risk zone Area I	-	Clade B	B3
MF6579	Illumina MiSeq	SAMN14314893	SRR11262086	1 167 579	M1	30.01.2017	cooked	High-risk zone Area I	-	Clade B	B3
MF6582	Illumina MiSeq	SAMN14314894	SRR11262084	1 379 603	M1	15.02.2017	cooked	High-risk zone Area II	-	Clade A	A-M1
MF6587	Illumina MiSeq	SAMN14314895	SRR11262083	1 350 241	M1	07.03.2017	cooked	Unknown	-	Clade B	B3
MF6588	Illumina MiSeq	SAMN14314896	SRR11262082	1 099 115	M1	17.03.2017	cooked	High-risk zone Area II	-	Clade B	B3
MF6589	Illumina MiSeq	SAMN14314897	SRR11262081	1 322 992	M1	22.03.2017	cooked	High-risk zone Area II	-	Clade B	B3
MF6590	Illumina MiSeq	SAMN14314898	SRR11262080	1 600 434	M1	29.03.2017	cooked	High-risk zone Area I	-	Clade B	B3
MF6591	Illumina MiSeq	SAMN14314899	SRR11262079	1 461 926	M4	24.03.2017	cooked	High-risk zone	-	Clade B	B2
MF6592	Illumina MiSeq	SAMN14314900	SRR11262078	1 237 734	M1	07.04.2017	cooked	High-risk zone Area I	-	Clade B	B3
MF6593	Illumina MiSeq	SAMN14314901	SRR11262077	1 468 623	M1	20.04.2017	cooked	High-risk zone Area I	-	Clade B	B3
MF6705	Illumina MiSeq	SAMN14314902	SRR11262076	1 254 502	M1	20.04.2017	cooked	Unknown	-	Clade B	B3
MF6706	Illumina MiSeq	SAMN14314903	SRR11262075	1 610 559	M1	15.05.2017	cooked	High-risk zone Area II	-	Clade B	B3
MF6707	Illumina MiSeq	SAMN14314904	SRR11262073	1 292 458	M1	16.05.2017	cooked	High-risk zone Area II	-	Clade A	A-M1
MF6709	Illumina MiSeq	SAMN14314905	SRR11262072	691 258	M1	02.06.2017	cooked	High-risk zone Area II	-	Clade A	A-M1
MF6710	Illumina MiSeq	SAMN14314906	SRR11262071	964 373	M1	15.06.2017	cooked	High-risk zone Area II	-	Clade B	B3
MF6797	Illumina MiSeq	SAMN14314907	SRR11262070	515 156	M1	30.06.2017	cooked	High-risk zone Area II	-	Clade B	B3
MF6799	Illumina MiSeq	SAMN14314908	SRR11262069	494 951	M1	20.07.2017	cooked	Unknown	-	Clade B	B3
MF6800	Illumina MiSeq	SAMN14314909	SRR11262068	972 245	M1	28.07.2017	cooked	High-risk zone Area II	-	Clade A	A-M1
MF6801	Illumina MiSeq	SAMN14314910	SRR11262067	1 277 801	M1	11.08.2017	cooked	High-risk zone Area I	-	Clade B	B3
MF6802	Illumina MiSeq	SAMN14314911	SRR11262066	967 404	M1	17.08.2017	cooked	High-risk zone Area II	-	Clade B	B3
MF6803	Illumina MiSeq	SAMN14314912	SRR11262065	1 346 734	M1	31.08.2017	cooked	High-risk zone Area II	-	Clade A	A-M1
MF6809	Illumina MiSeq	SAMN14314913	SRR11262064	1 064 322	M1	08.09.2017	cooked	High-risk zone Area I	-	Clade B	B3
MF6810	Illumina MiSeq	SAMN14314914	SRR11262062	1 333 231	M1	15.09.2017	cooked	High-risk zone Area I	-	Clade B	B3
MF6811	Illumina MiSeq	SAMN14314915	SRR11262061	649 115	M1	15.09.2017	cooked	High-risk zone Area I	-	Clade B	B3
MF6817	Illumina MiSeq	SAMN14314916	SRR11262060	1 079 103	M1	09.11.2017	cooked	High-risk zone Area II	-	Clade B	B3
MF6818	Illumina MiSeq	SAMN14314917	SRR11262059	864 840	M1	15.11.2017	cooked	High-risk zone Area II	-	Clade A	A-M1

MF6819	Illumina MiSeq	SAMN14314918	SRR11262058	1 117 102	M1	15.11.2017	cooked	High-risk zone Area II	-	Clade A	A-M1
MF6820	Illumina MiSeq	SAMN14314919	SRR11262185	1 333 962	M1	15.11.2017	cooked	High-risk zone Area II	-	Clade A	A-M1
MF6821	Illumina MiSeq	SAMN14314920	SRR11262184	1 462 552	M1	15.11.2017	cooked	High-risk zone Area II	-	Clade A	A-M1
MF6822	Illumina MiSeq	SAMN14314921	SRR11262183	1 773 315	M1	15.11.2017	cooked	High-risk zone Area II	-	Clade A	A-M1
MF6823	Illumina MiSeq	SAMN14314922	SRR11262182	1 854 842	M1	15.11.2017	cooked	High-risk zone Area II	-	Clade B	B3
MF6824	Illumina MiSeq	SAMN14314923	SRR11262181	1 732 800	M1	15.11.2017	cooked	High-risk zone Area II	-	Clade B	B3
MF6836	Illumina MiSeq	SAMN14314924	SRR11262179	1 533 267	M1	13.10.2017	cooked	High-risk zone Area I	-	Clade A	A-M1
MF6837	Illumina MiSeq	SAMN14314925	SRR11262178	1 518 498	M1	13.10.2017	cooked	High-risk zone Area I	-	Clade B	B3

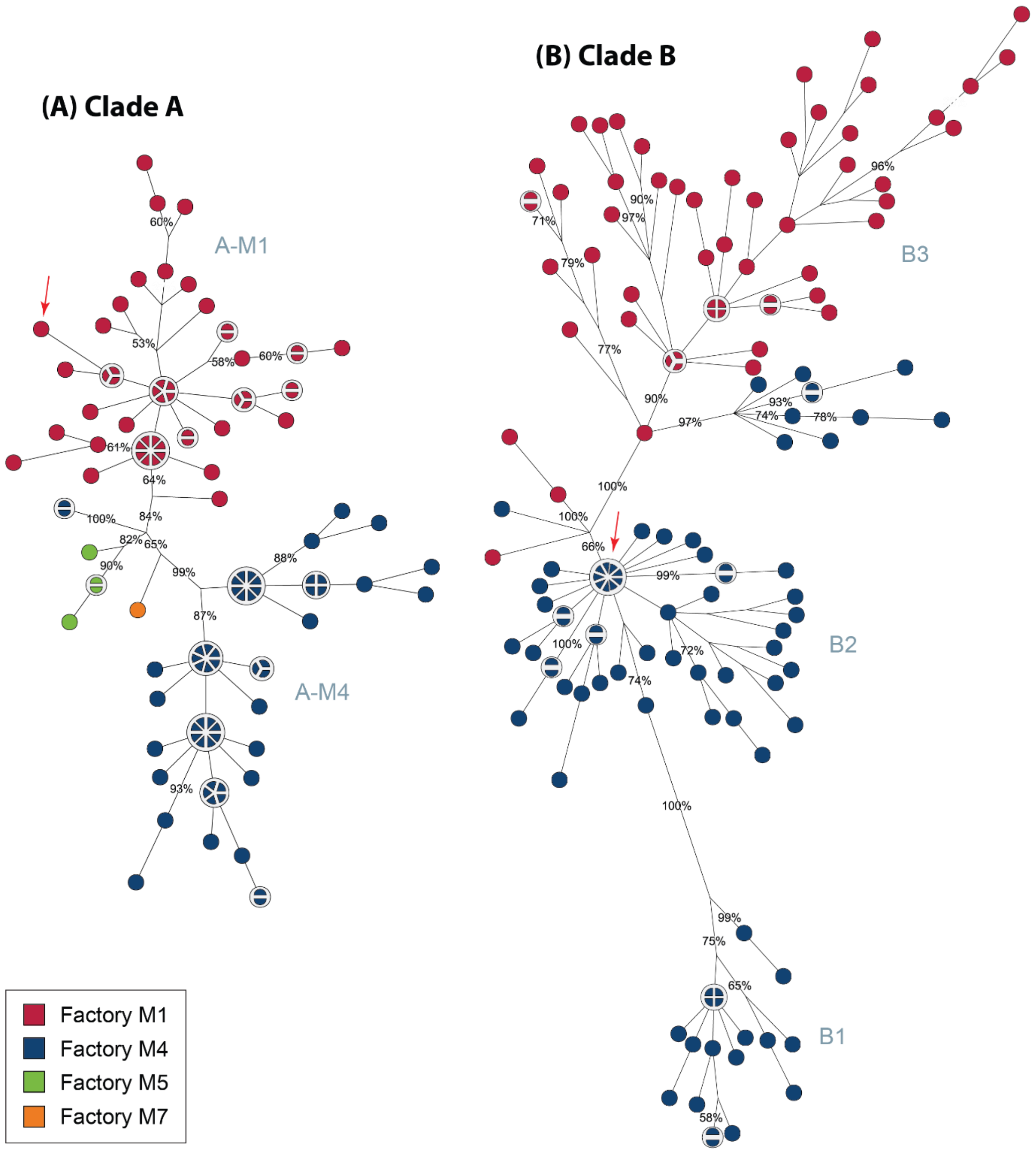

Supplemental Figure S1

Maximum parsimony phylogenetic trees based on SNP alignments generated for genomes belonging to **(A)** Clade A (based on 121 SNPs) and **(B)** Clade B (based on 331 SNPs). The reference genomes used were the complete genome sequences of **(A)** isolate MF4562 (GenBank accession no. CP025442) and **(B)** isolate MF4697 (GenBank accession no. CP025438), belonging to each respective clade. The position of the reference genome in each tree is indicated with an arrow. The area of each circle is proportional to the number of isolates represented and the factory of origin for each isolate is indicated by the color. Branch lengths were scaled using square root scaling. Labels on interior branches represent bootstrap resampling support values, showing only values >50%. Labels next to selected clusters refer to the subclusters described in the text.

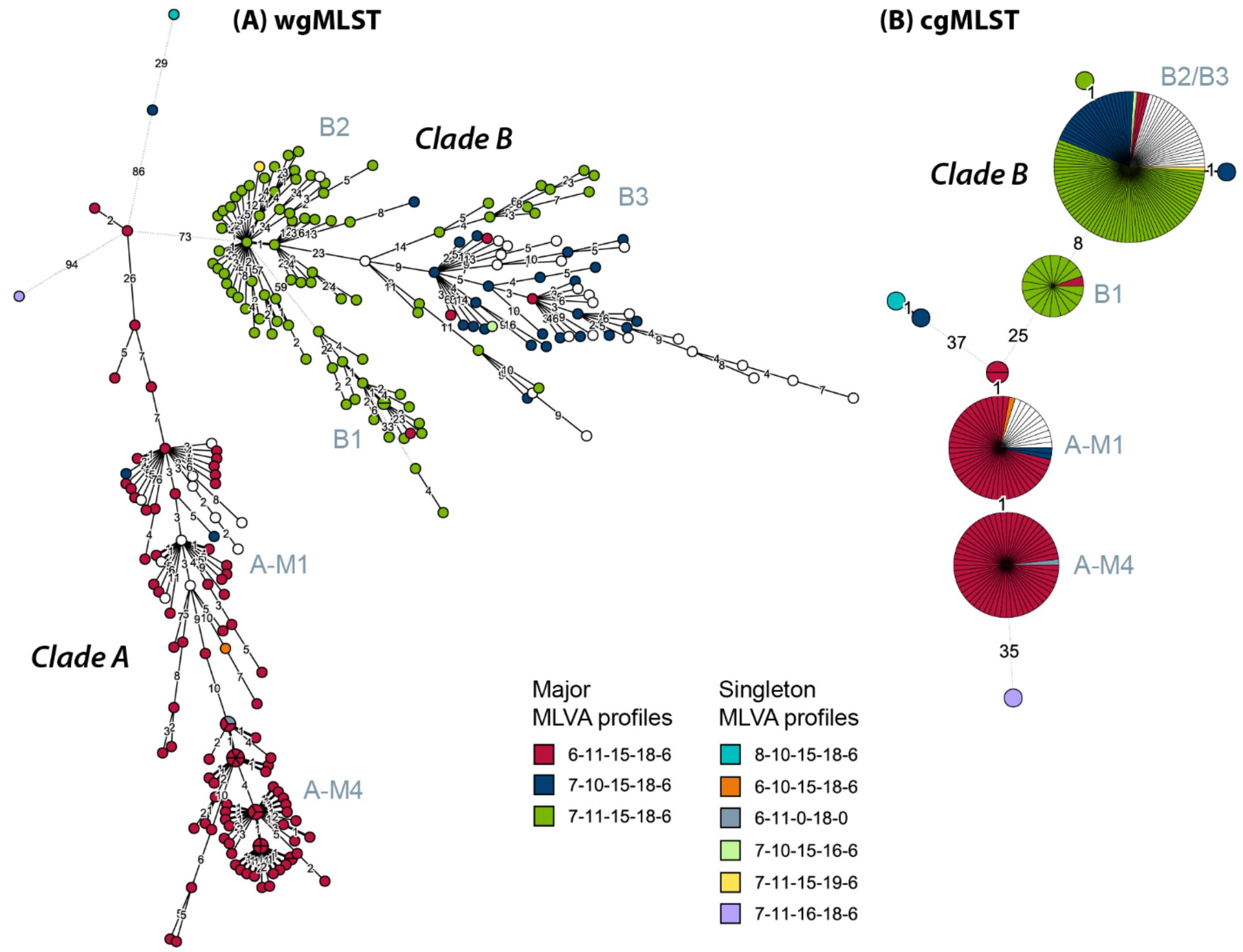

Supplemental Figure S2

Minimum spanning trees based on **(A)** wgMLST and **(B)** cgMLST alleles, coloured according to MLVA profile. Isolates represented by white nodes have not been typed using MLVA. Branch lengths were scaled using **(A)** logarithmic and **(B)** square root scaling.

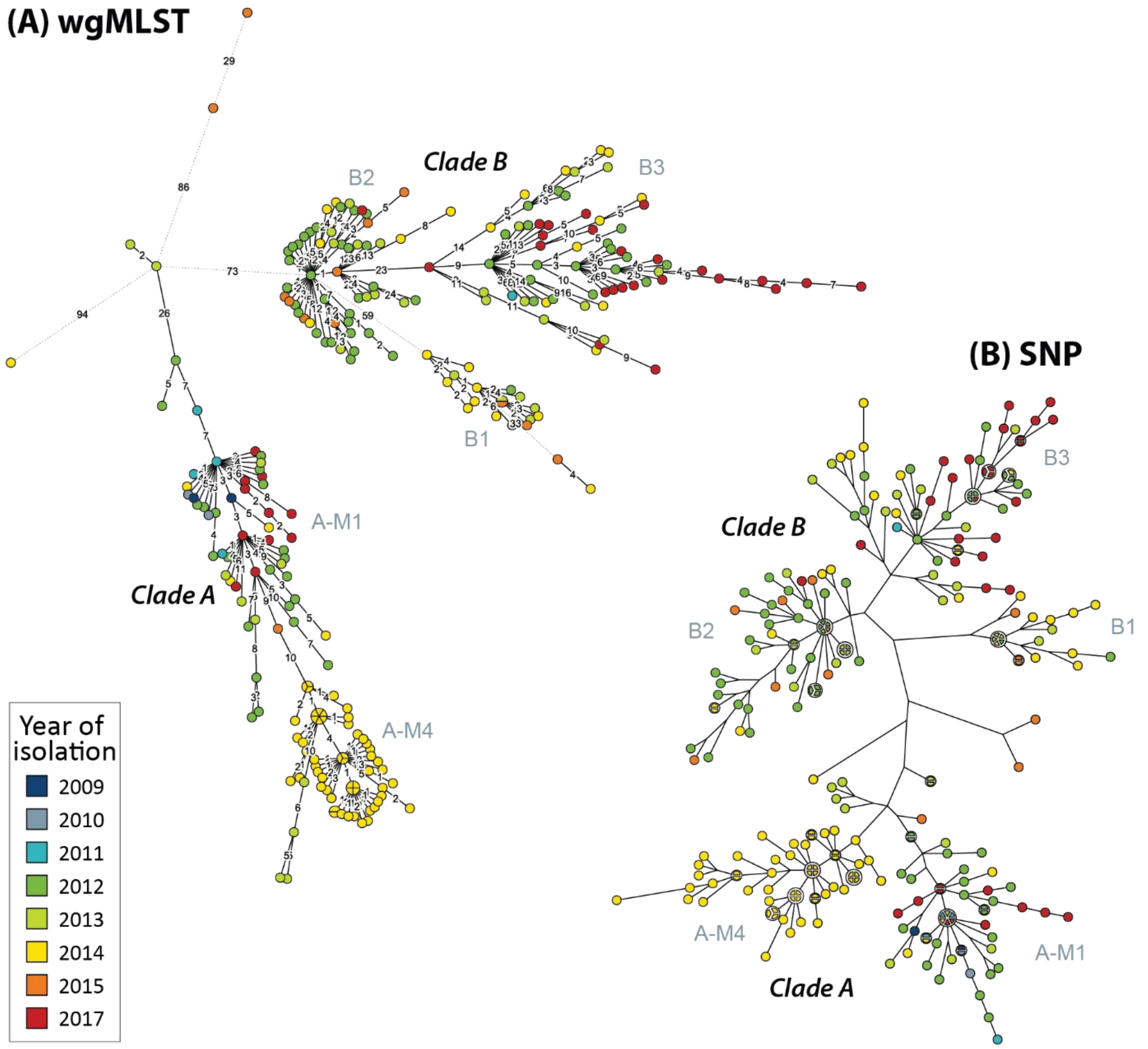

Supplemental Figure S3

Phylogenetic trees for ST9 isolates from Norwegian meat processing industry coloured by year of isolation. **(A)** Minimum spanning tree based on wgMLST alleles, with logarithmic scaling of branches. **(B)** Maximum parsimony tree based on the SNP alignment, with square root scaling of branches.

Supplemental Figure S4 (next page)

The maximum clade credibility tree shown in Figure 3, with posterior probability values for each split and node labels containing strain names.

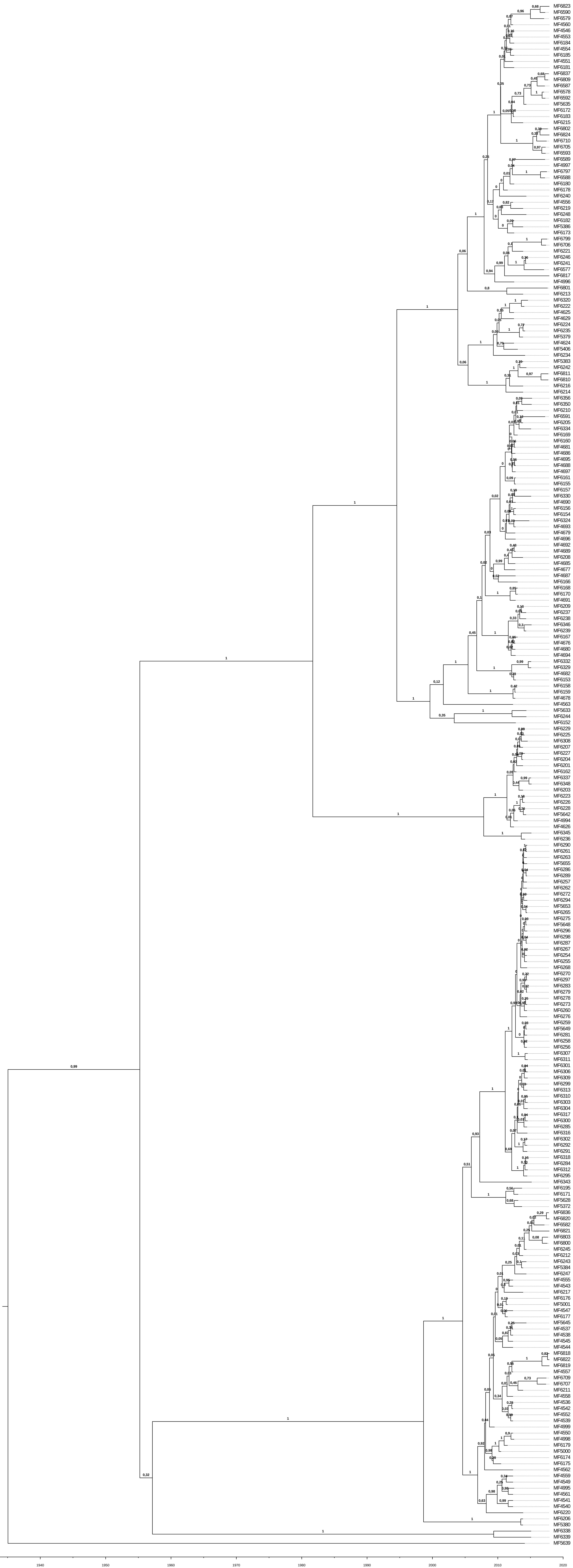

MF5639
MF6338
MF6339
1940
1950
1960
1970
1980
1990
2000
2010
2020
